# Genomic variations leading to alterations in cell morphology of *Campylobacter* spp

**DOI:** 10.1038/srep38303

**Published:** 2016-12-02

**Authors:** Diane Esson, Alison E. Mather, Eoin Scanlan, Srishti Gupta, Stefan P. W. de Vries, David Bailey, Simon R. Harris, Trevelyan J. McKinley, Guillaume Méric, Sophia K. Berry, Pietro Mastroeni, Samuel K. Sheppard, Graham Christie, Nicholas R. Thomson, Julian Parkhill, Duncan J. Maskell, Andrew J. Grant

**Affiliations:** 1 Department of Veterinary Medicine, University of Cambridge, Madingley Road, Cambridge, UK; 2 Wellcome Trust Sanger Institute, Wellcome Trust Genome Campus, Hinxton, Cambridge, UK; 3 Department of Chemical Engineering and Biotechnology, University of Cambridge, New Museums Site, Pembroke Street, Cambridge, UK; 4 The Milner Centre for Evolution, Department of Biology and Biotechnology, University of Bath, Claverton Down, Bath, UK; 5 The London School of Hygiene and Tropical Medicine, London, UK

## Abstract

*Campylobacter jejuni,* the most common cause of bacterial diarrhoeal disease, is normally helical. However, it can also adopt straight rod, elongated helical and coccoid forms. Studying how helical morphology is generated, and how it switches between its different forms, is an important objective for understanding this pathogen. Here, we aimed to determine the genetic factors involved in generating the helical shape of *Campylobacter*. A *C. jejuni* transposon (Tn) mutant library was screened for non-helical mutants with inconsistent results. Whole genome sequence variation and morphological trends within this Tn library, and in various *C. jejuni* wild type strains, were compared and correlated to detect genomic elements associated with helical and rod morphologies. All rod-shaped *C. jejuni* Tn mutants and all rod-shaped laboratory, clinical and environmental *C. jejuni* and *Campylobacter coli* contained genetic changes within the *pgp1* or *pgp2* genes, which encode peptidoglycan modifying enzymes. We therefore confirm the importance of Pgp1 and Pgp2 in the maintenance of helical shape and extended this to a wide range of *C. jejuni* and *C. coli* isolates. Genome sequence analysis revealed variation in the sequence and length of homopolymeric tracts found within these genes, providing a potential mechanism of phase variation of cell shape.

*Campylobacter* spp. are the most common cause of bacterial food-borne diarrhoeal disease worldwide^1^. A major route of human infection is through the consumption of contaminated meats, especially chicken, and milk or through faecal-oral transmission^2^,^3^,^4^,^5^,^6^. A fundamental property of most *Campylobacter spp.* is that its cells are helical, and that these can change their shape to become rod or coccoid-shaped^7^. Helical cell shape is relatively rare within bacterial species and is thought to confer various colonisation and pathogenic advantages for *Campylobacter jejuni* and *Campylobacter coli*, including improved translocation across mucus membranes by facilitating a ‘corkscrew’-like motility^7^,^8^,^9^ and a loss of helical cell shape is linked to attenuated chick colonisation^10^.

To better understand how helical-shaped *Campylobacter* spp. maintain their cell shape and thus any associated colonisation, pathogenic or other advantage, it is first important to identify the genes and proteins involved. While the *C. jejuni* genome encodes cytoskeleton regulatory elements MreB, RodA and FtsZ, important for maintaining rod morphology in many bacteria^11^,^12^,^13^,^14^,^15^,^16^, these proteins alone are not sufficient to generate helical and curved bacterial morphologies^11^,^17^,^18^. Mechanisms for generating helical and curved morphologies have been identified in other bacterial species, including the proteins encoded by the genes *ccrp59, ccrp1143, csd1, csd2, csd3* and *ccmA* in *Helicobacter pylori*^19^,^20^ and *creS* in *Caulobacter crescentus*^21^. So far, the only genes known to be involved in determination of the helical cell shape of *C. jejuni* are *pgp1* and *pgp2*, and their protein products are peptidoglycan (PG) peptidases that are important for PG synthesis^9^,^10^,^22^. When either of the *pgp1* or *pgp2* genes were mutated, the muropeptide profile radically changes and helical cell shape could not be maintained^9^,^10^.

Pgp1 is a DL-carboxypeptidase which cleaves monomeric PG tripeptides to dipeptides^9^. Deletion or over-expression of *pgp1* results in rod-shaped morphologies. Thus, the proper ratio of monomeric tripeptides to dipeptides may be required for proper shape determination^9^. Loss of *pgp1* affects pathogenic attributes such as chicken colonisation, biofilm formation, motility and activation of host inflammatory mediators^9^. Pgp2 is an LD-carboxypeptidase cleaving PG tetrapeptides to tripeptides, which provide the substrate for the DL-carboxypeptidase Pgp1^10^. Unlike *pgp1, pgp2* is not restricted to helical and vibroid bacteria and is conserved in both Gram negative and Gram positive bacteria^10^.

In this study, we aimed to identify novel genetic determinants of *C. jejuni* helical cell shape. To do this, a *C. jejuni* signature-tagged transposon mutant (STM) library^23^ was screened by light microscopy for altered cell morphologies. Of 1,933 STMs screened, 132 revealed changes in cell shape, including rod and short helical morphologies. However, transposon (Tn) insertion sites could not account for these cell shape differences. Instead, whole genome sequencing (WGS) was used to investigate genomic variation between STMs. In addition, we found a direct correlation between cell and colony morphologies, enabling the isolation and sequencing of rod-shaped bacteria from wild-type (WT) populations. All sequenced rod-shaped *C. jejuni* isolates contained a single nucleotide polymorphism (SNP) or small insertion or deletion (INDEL) in the genes encoding the peptidoglycan remodelling enzymes Pgp1 or Pgp2.

## Results and Discussion

### Identification and isolation of rod-shaped *C. jejuni* mutants

**Figure 1:**
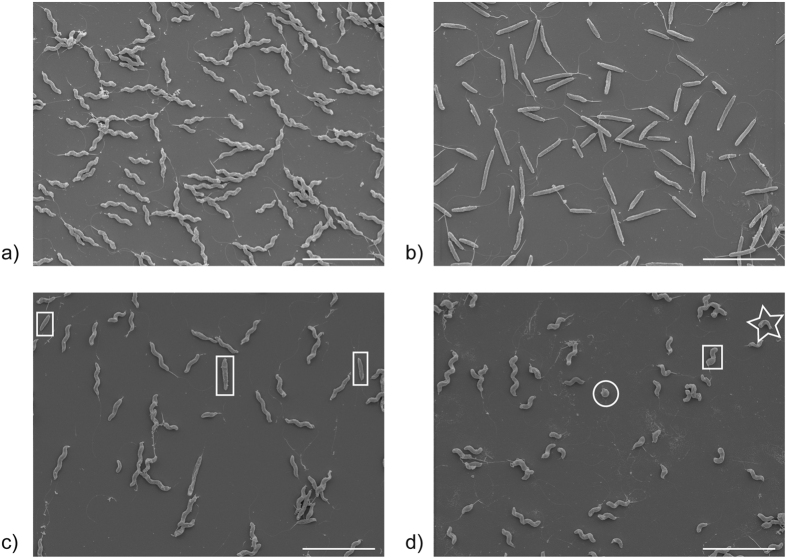
Scanning electron micrographs of observed *C. jejuni* morphologies. (**a**) helical, (**b**) rod, (**c**) rod-and-helical and (**d**) short helical *C. jejuni* M1 isolates. Rod bacteria in (**c**) are boxed. Short helical cultures (**d**) include S-shaped (box), crescent (star) and/or coccoid (circle) bacteria. Scale bars represent 5 μm.

Tn mutant libraries are a useful tool for screening a range of phenotypes derived from random mutation. We screened a pre-existing STM Tn library constructed in *C. jejuni* strain M1^23^ by light microscopy, with the aim of identifying bacteria with altered cell morphologies. STMs analysed in the screen were derived from 45 different signature tags, with each tag comprising 10–50 STMs. Of 1,933 Tn mutants cultured and screened individually, 89 were exclusively rod-shaped, 22 had mixed rod and helical cell morphologies and 25 showed an exclusively “short-helical” morphology, which included a mixture of helical, S-shaped, crescent and coccoid forms (Fig. 1).

The STM Tn mutants screened were generated as 1,933 independent and random mutation events but, the observed morphologies had a seemingly non-random distribution within the library: 109 of the 136 non-helical Tn mutants belonged to only seven signature tags (Fig. S1). We used linker PCR and plasmid rescue to determine the chromosomal location of the Tn insertion sites within 49 non-helical Tn mutants, and found that the pattern of non-helical morphologies did not correlate clearly with particular Tn insertion sites, suggesting that the observed cell morphologies were not the result of the primary Tn mutation event. To investigate this in more detail, we decided to test some of the genes mutated in the Tn library for their role in cell shape determination. We chose three genes for this initial analysis that were similar to genes from other bacteria that are involved in cell shape determination. These were *CJM1_0159* which encodes a predicted coiled-coil region also seen in crescentin, a protein responsible for curvature in *C. crescentus*^21^, and *CJM1_0631* and *dtpT* which encode large, transmembrane di-/tripeptide transporters that we predicted were either important for cell wall integrity or transported substrates necessary for PG synthesis. We mutated these genes in *C. jejuni* by allelic-replacement and showed that this did not consistently result in rod morphology in the WT background. Taken together, the data indicated that the Tn mutation event was not responsible for rod shape cells in the library and that therefore another source of variation may be responsible for the loss of helical cell shape.

Without a clear pattern of Tn insertion sites to account for the different morphologies within the library, we hypothesised that there was another source of variation within the non-helical STMs. Since STM morphologies were maintained throughout the cell cycle and across multiple generations, we further hypothesised this was due to a genetic mutation, as opposed to differences in genetic regulation.

Pulsed-field gel electrophoresis (PFGE), a highly discriminatory technique used for distinguishing bacterial strains^24^,^25^ was performed to investigate whether major chromosomal rearrangements could account for the difference between rod-shaped STMs and the WT. No major chromosomal rearrangements could be detected by pulse-field gel electrophoresis.

In lieu of major chromosomal rearrangement, we performed WGS to search for polymorphisms that might explain the phenotypes. In total, 66 rod-shaped Tn mutants, 6 short-helical shaped Tn mutants and 15 Tn mutants showing mixed rod and helical morphologies were selected and sequenced alongside the WT parent and 6 helical Tn mutants as controls.

**Figure 2:**
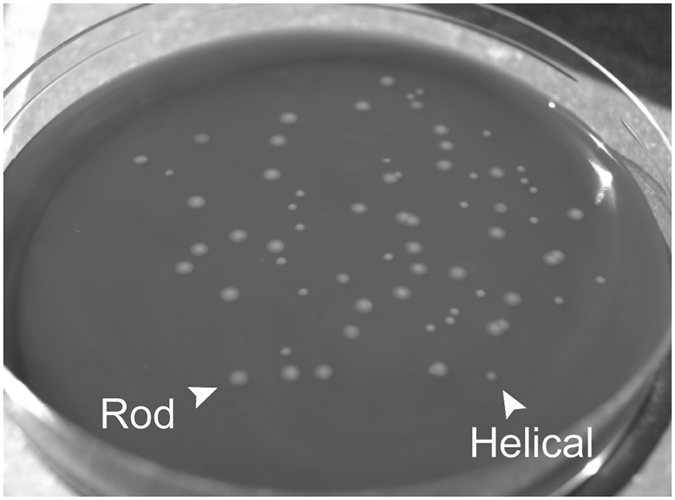
Helical and rod *C. jejuni* have distinct colony morphologies. Colony growth of a mixed population of helical and rod *C. jejuni* M1 WT bacteria. The plate shows the, flattened and often larger colonies of rod bacteria and the shiny and rounded colonies of helical bacteria.

**Figure 3:**
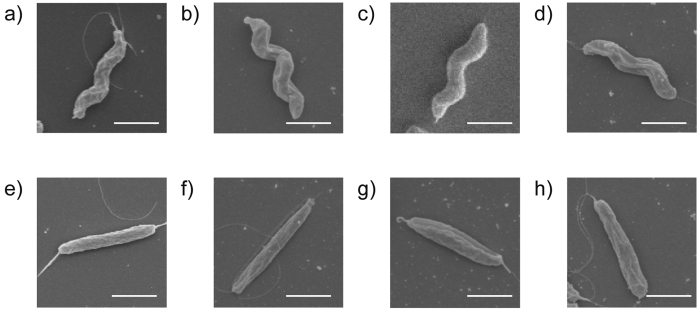
Scanning electron micrographs of helical and rod WT *C. jejuni* isolates. Helical isolates from *C. jejuni* strains (**a**) M1, (**b**) 81116, (**c**) 81–176 and (**d**) NCTC11168 and rod isolates from (**e**) M1, (**f**) 81116, (**g**) 81–176 and (**h**) NCTC11168. Scale bars represent ~1 μm.

Importantly, we observed that, although the WT M1 strain is characterised by helical morphology, our WT M1 laboratory strain (believed to have been passaged only twice since original isolation) contained a mixture of helical and rod-shaped cells. This finding is consistent with the discovery of a proportion of rod-shaped bacteria within the predominantly helical targeted deletion strains already mentioned. Following this discovery, we sought to isolate pure populations of the helical and rod-shaped bacteria and noticed a clear association between cell and colony morphologies. Helical bacteria formed shiny, rounded colonies on MH agar whereas rod-shaped bacteria formed dull, flat and often larger colonies (Fig. 2). This association was confirmed by light microscopy for over 150 colonies. We hypothesise that the different colony morphologies may be due to a discrepancy in the packing of helical versus rod cells within the colony^26^, as observed in cell shape mutants of other bacterial species^18^. We used colony purification to isolate helical and rod-shaped populations from *C. jejuni* strains M1, NCTC11168, 81116 and 81–176 (Fig. 3), as well as from the isogenic M1 mutant *CJM1_0159*. A selection of helical and rod-shaped WT isolates from these four *C. jejuni* strains was included in the WGS analysis. We have summarized the string of methods applied and conclusions made in the process of the STM analysis, as described above, in Fig. S2.

To determine whether *C. jejuni* morphological changes could be observed during *in vitro* growth, we performed serial passages of single colonies on MH agar and screened each passage for cell shape mutants. Specifically, we identified rod-shaped bacteria in passages of helical bacteria, and for helical-shaped bacteria in passages of rod bacteria. We screened over 40,000 colonies from four passages of both helical and rod WT lineages and identified four rod-shaped mutants from the helical lineages. These rod-shaped mutants were from the first passage of isolate H2 (H2_P1_R1, where H2 = helical isolate 2, P1 = first passage and R1 = rod 1), the second passage of isolate H5 (H5_P2_R1 and H5_P2_R2) and the fourth passage of isolate H5 (H5_P4_R1). No helical-shaped revertants from the rod lineages were identified. The four rod-shaped clones, each with a helical isolate from the same lineage and passage number as a control, were analysed by WGS. This screen for cell shape variants was also used to determine the rate of helical-to-rod morphological change in *C. jejuni* M1, which we calculated as a frequency of 7.7 × 10^−4^, or 7.25 × 10^−5^ phenotypic variants/division (Table S1).

### Genome sequenced *C. jejuni* isolates were genetically distinct

The genome sequence data from 133 helical and rod Tn mutants and WT isolates were analysed for the presence of single nucleotide polymorphisms (SNPs) or small insertions and deletions (INDELs) (Table S2) and changes in the number of bases in documented phase variable regions (PVRs) (Table S3).

Phase variation (PV) is a common source of genetic and phenotypic variation in many bacteria, including *C. jejuni*^27^,^28^,^29^. PV enables a stochastic ‘on’ and ‘off’ switching of genes in bacteria, providing population diversity that may promote immune evasion or survival in varying environmental conditions^26^ or if the bacterium inhabits more than one niche^30^. Regions of the bacterial genome that are particularly prone to these reversible mutations are known as PVRs and include simple sequence repeats, inverted repeats, gene duplications and methylation sites. Homopolymeric tracts (HTs) are highly susceptible to slipped-strand mispairings, which alter the length of the tracts and generate frameshift mutations during DNA replication and repair^26^,^31^. The frameshift mutations cause a change to the downstream amino acid sequence and almost always result in premature stop codons. In this way, PVRs are able to randomly switch genes ‘on’ and ‘off’ and stochastically regulate gene expression^26^. Although these mutations are heritable, they are also often reversible. This is usually evident by a variety of HT lengths of a PVR within a population^27^,^28^ or observed directly by bacterial passage^32^,^33^. The rate of PV is generally greater than that of spontaneous mutation and is typically calculated to 10^−4^ mutations/division or more^26^,^28^,^34^. Rates of PV can differ depending on environmental conditions and/or whether the genotypic switch is from ‘on-to-off’ or ‘off-to-on’ and these rates can vary up to 10-fold^28^,^34^,^35^,^36^,^37^.

The propensity of *C. jejuni* to undergo PV^29^ may be exacerbated by the absence of a functional mismatch repair (MMR) system in this species^38^. MMR systems have been found to protect bacterial genomes from slipped-strand mispairing^39^,^40^,^41^ and it has been postulated that the lack of this repair mechanism in *C. jejuni* makes its genome more susceptible to PV^38^. Indeed, MMR mutants in other species show increased mutation rates^42^,^43^. Alternatively, higher rates of PV in *C. jejuni* could be due to inaccuracies of the *C. jejuni* DNA polymerase, as has also been suggested for *H. pylori*^44^.

In *C. jejuni*, PVRs have been identified in capsule, LOS and flagellin glycosylation loci as well as a range of loci of unknown function^4^,^27^,^28^,^45^. Most identified PVRs in *C. jejuni* are within polyG:C tracts of seven or more nucleotides in length^26^,^27^,^45^. However, PV in *C. jejuni* has also been observed in polyA:T tracts and in HTs as short as two nucleotides^27^,^32^, demonstrating that this phenomenon is not limited by nucleotide or tract length. However, investigations into variable polyA:T tracts are limited by their density within the *C. jejuni* genome, which is roughly 70% AT rich^27^. Considering all these complexities of PV analysis, it is reasonable to hypothesise that PV in *C. jejuni* may occur in more regions than currently recorded.

The WGS demonstrated the fluidity and susceptibility to mutation of the *C. jejuni* genome. There were numerous SNPs and INDELs that were distinct from the GenBank reference genome of the same strain (CP001900.1). Interestingly, genetic differences were observed in isolates from the same laboratory stock, the same isolates following *in vitro* passage and from isolates subjected to genetic manipulation. Many mutations identified throughout the *C. jejuni* genomes fell within HTs of a range of lengths and nucleotide sequence. As previously described, slipped strand mispairing at runs of Cs or Gs has been described in *C. jejuni*^27^. Although rarely classified as a mechanism of PV in *Campylobacter*, we present evidence here that runs of As or Ts are also susceptible to length variation, suggesting that these regions are particularly susceptible to mutation and that PV in *Campylobacter* should be re-evaluated. However, whether these mutations occur naturally within *C. jejuni* populations, due to an error prone DNA polymerase, a lack of DNA repair mechanisms and/or the instability of HTs, or whether the mutations emerge from stresses induced by the laboratory environment and genetic manipulation remains to be determined. For example, differences within the STM library could be derived from differences in growth or transformation micro-environments while the STM library was being made. Collectively, the presence of these mutations warns against assumptions that isolates within any *C. jejuni* population are genetically identical (also addressed in refs 46, 47, 48, 49) or that any site-directed genotypic changes to *C. jejuni* are responsible for observed phenotypic changes. To overcome this uncertainty during phenotypic characterisation, the precedent should be maintained that *C. jejuni* isolates should be assessed by genome sequencing, by appropriate complementation analyses of mutants, and/or by performing laboratory manipulations and physiological assays on a panel of isolates.

### Mutations in *pgp1* or *pgp2* were present in all rod-shaped *C. jejuni* laboratory isolates

**Figure 4:**
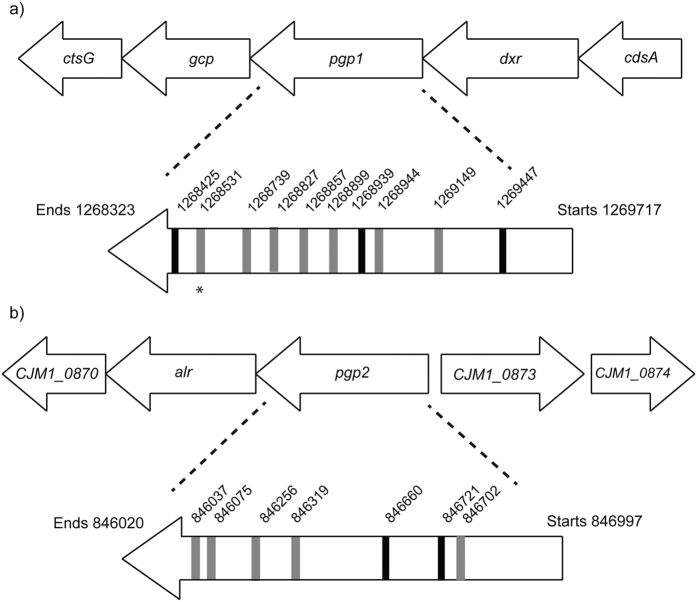
Mutations observed in *pgp1* and *pgp2*. Base locations of mutations in (**a**) *pgp1* and (**b**) *pgp2* are in reference to the *C. jejuni* M1 genome (CP001900.1)^4^. Black horizontal bars indicate SNPs; grey horizontal bars indicate INDELs. All INDELs are located in homopolymeric tracts. Asterisk (*) below *pgp1* indicates the 8-A tract, in which mutations were observed in more than one *C. jejuni* strain.

Every rod-shaped Tn mutant from the M1 STM library and every rod-shaped isolate from WT strains M1, 81116, 81–176 and NCTC11168 had a SNP or INDEL within the PG peptidase-encoding genes *pgp1* or *pgp2* (Fig. 4. and Table 1). Rod-shaped bacteria isolated from the mixed rod-and-helical Tn mutants also contained a SNP or INDEL within *pgp1* or *pgp2* (as determined by PCR and Sanger sequencing) (Fig. 4. and Table 1). No mutations in either of these genes were observed in any of the helical isolates. This strict correlation between the rod-shaped morphology and mutations in these genes was confirmed by site-directed mutagenesis (SDM) of a selection of rod STMs and rod WT isolates. SDM was performed to lock one of the variable homopolymeric tracts in *pgp1* and *pgp2* into its ‘on’ length. The rod STM isolate adopted a helical morphology after the incorporation of *pgp1_ON* (Fig. S3) and all three tested rod STMs adopted a helical morphology after the incorporation of *pgp2_ON* (Fig. S4).

Mutations in *pgp1* and *pgp2* were in a wide array of base locations (Fig. 4) and their predicted effects on translation included single amino acid changes and 4 bp truncations – all of which were sufficient to cause a predicted loss of function of these proteins. Function was tested by analysis of the cell wall muropeptides of rod-shaped and helical isolates, using High-Performance Liquid Chromatography (HPLC) (Fig. S5, and Table S4). These analyses demonstrated that rod-shaped *pgp1* mutants in both the M1 and 81116 backgrounds have similar muropeptide profiles, and that these are distinct from the muropeptide profiles from their respective helical WT cells. Likewise, muropeptide profiles of rod-shaped *pgp2* mutants in the M1 and 81116 backgrounds were similar to each other and distinct from WT cells. The muropeptide compositions are also similar to those of *pgp1* mutants, *pgp2* mutants or WT in the 81–176 background^9^,^10^, except that the putative dipeptide (peak 3) and putative acetylated tetra-tetrapeptide (peak 12) fractions are greater in the 81116 and M1 *pgp1* mutants than in the 81–176 *pgp1* mutant. These profiles also expand our understanding of muropeptide compositions of helical versus rod-shaped bacterial species.

Combined, these data demonstrate that many different mutations can affect the functionality of Pgp1 and Pgp2. Interestingly, the most frequent site of mutation within *pgp1* was an HT consisting of 8 As towards the 3′ end of the gene. This HT had 7 or 9 As in many of the rod-shaped isolates in the M1, 81116 and 81–176 backgrounds, resulting in a truncation that is predicted to remove approximately 14% of the WT protein. The rate of morphological change is greater than that of the spontaneous mutation (Table S1), so we speculated that there may be phase variable control of cell shape.

To further investigate this hypothesis, we conducted another experiment looking for rod-shaped bacteria in passages of helical bacteria, and for helical-shaped bacteria in passages of rod bacteria. In this experiment, the starting helical isolate was M1cam (Table S5) and the rod isolate was M1 ‘R2’ containing the HT 7A (Table S5). 112 spontaneous rod derivatives were identified from 375,000 screened colonies. Over 525,000 colonies were screened looking for helical derivatives from the rod-shaped starting bacteria without detection of changes in colony morphology. Next we performed WGS on genomic DNA obtained from each of these colonies and mapped the reads to the M1cam (CP012149) and identified the SNPs and INDELS. We discovered that the helical to rod isolates R’50, R’82 and R’88 all had normal CJM1cam_0872 (*pgp1*), and all had a frameshift caused by a 2-base insertion in the same position in CJM1cam_1302 (*pgp2*) (Fig. 4, Table 1). All other helical to rod isolates had normal CJM1cam_1302 (*pgp2*), and all had a frameshift caused by a single base deletion in the same position in CJM1cam_0872 (*pgp1*) (Fig. 4, Table 1). This screen was also used to determine the rate of helical-to-rod morphological change in *C. jejuni* M1, under these experimental conditions, which we calculated as a frequency of 2.1 × 10^−4^ (Table S1). This frequency is similar to the frequency that we calculated with a different experimental procedure (Table S1), and the differences are likely to be due to differences in the experimental procedure, and the estimation of the number of colonies screened.

These data, combined with the data from the earlier experiments, demonstrate that the helical-to-rod morphological switch occurs repeatedly in standard laboratory growth conditions. It remains possible that the rod-to-helical switch is also occurring but at a lower rate. Alternatively, different environmental pressures may be necessary to observe the rod-to-helical switch.

### Clinical and environmental *C. jejuni* and *C. coli* isolates contain length variants of the 8-A tract in *pgp1*

Next, we examined whether HT length polymorphisms in *pgp1* and *pgp2* also existed in a range of *C. jejuni* isolates from various environmental and clinical sources. We investigated the presence and allelic variances of *pgp1* and *pgp2* in 859 genomes of *C. jejuni* and *C. coli*. The genomes were from a wide range of isolates: 192 from clinical, agricultural and wild bird sources^48^, 319 from multiple stages of poultry processing, including farms, abattoirs and retail chicken meat^50^ and 348 from clinical human cases^51^. Analysis of these genomes revealed that all isolates contained *pgp1* and *pgp2*, suggesting that these genes are core to *C. jejuni* and *C. coli*. Furthermore, although no length variation was detected at the 4-A HT in *pgp2* (where variation had been observed in the *C. jejuni* Tn mutant library), 29 genomes (3% of isolates) contained length variation at the 8-A HT in *pgp1* (Table 2).

**Figure 5:**
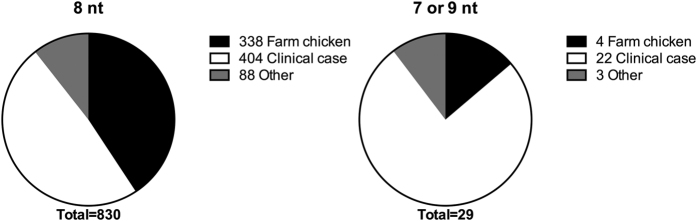
Prevalence of poly-A tract lengths in the *pgp1* allele of *Campylobacter spp.* isolated from various sources. The two distributions show a significant discrepancy (p = 0.0083; χ^2^ for goodness of fit test; χ^2^ = 9.571, d.f. = 2).

The morphology of a selection of these *C. jejuni* and *C. coli* isolates was investigated by light microscopy. All isolates with 7-A or 9-A length polymorphisms at the 8-A HT in *pgp1* were rod-shaped. Furthermore, a large proportion of isolates with the 7-A or 9-A HTs were from clinical sources (Fig. 5). Additionally, a recent report^22^ described the complete genome sequence of *C. jejuni* RM1285, a rod-shaped morphological variant, an environmental isolate derived from the exudate of a commercial chicken breast acquired from a retail store. RM1285 was reported to have a single A deletion at the 1,187^th^ nucleotide of *pgp1*, that resulted in a 62-amino acid truncation of the Pgp1 protein^22^. We have identified that the A deletion occurs in the 8-A HT described above. Together, these data suggest that HT length variation in *pgp1* may not simply be an artefact of laboratory conditions or manipulation and can affect the cell shape of *C. jejuni* and *C. coli* in the host and natural environment. However, it should be noted that none of our isolates, and we presume RM1285^22^, were screened directly from the primary source and each isolate had undergone a passage in the laboratory, therefore we cannot rule out the possibility that the change in the HT occurred during this growth step.

## Conclusions

Cell shape is a defining characteristic of bacterial species and members of a particular species normally retain a constant morphology for generation after generation. Yet functional questions pertaining to how bacteria have particular shapes, and what evolutionary advantage being a particular shape confers, have been largely neglected until recently.

In this study, an STM library screen was performed to identify random mutants of *C. jejuni* with morphological changes. None of the genes disrupted by Tns were found to be responsible for helical cell shape. Instead, through extensive genome sequence analysis, we found that the observed morphological changes in the *C. jejuni* M1 STM library were explained by background mutations (SNPs or INDELS) in either of the carboxypeptidase genes *pgp1* or *pgp2*, independent of the Tn insertions.

Reviewing the method used to generate the STM library, there were three stages in which background mutations could have emerged. The first was the growth of the WT population used to extract the genomic DNA, subsequently used in *in vitro* transposition. The second stage was the growth of the WT population used for natural transformation with the transposed DNA. The third stage was the growth of the transformants, prior to making frozen stocks. In any of these stages, a random mutational event could have occurred and proliferated to generate a non-helical STM. Considering the density of rod STMs within certain tags, with many STMs containing the same genomic variation, it is likely the rod-causing mutations in signature tags 50, 51, 53, 54, 55 and 57 occurred in the first or second stage. Mutations generated in these early stages would allow a single change to propagate throughout an entire tag or group of tags generated on the same day.

The discovery that both *pgp1* and *pgp2* are present in every genome from a collection of laboratory, environmental, wild bird, poultry and clinical *C. jejuni* and *C. coli* isolates strongly suggests *pgp1* and *pgp2* are core to these two helical *Campylobacter* species. Furthermore, the novel discovery that the 8-A HT in *pgp1* (AT PPVR3) is variable within this collection of *C. jejuni* and *C. coli* isolates, the recent study by Gunther *et al*.^22^, and the fact that indels in *pgp1* consistently correlates to a rod morphology when lengthened or shortened, emphasises the role of stochastic, heritable mutation in regulating *Campylobacter* cell shape in a range of environmental and host conditions.

**Figure 6:**
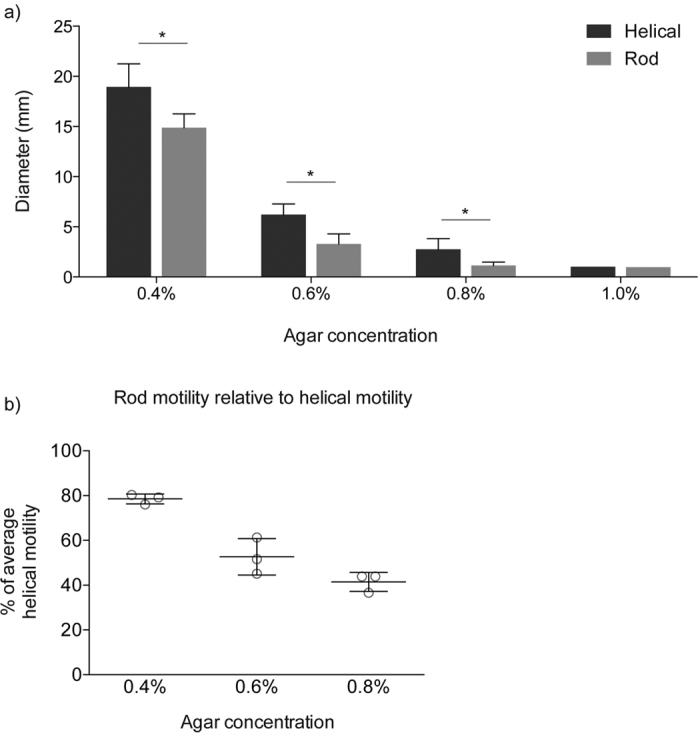
Motility of helical and rod WT *C. jejuni* M1 isolates. (**a**) Average motility of helical and rod WT *C. jejuni* M1 isolates in 0.4%, 0.6%, 0.8% and 1.0% (w/v) select agar. Motility for all isolates decreased with increasing agar concentration until isolates were effectively non-motile through 1.0% agar (all isolates measured 1 mm diameter, roughly equivalent to the original pipette stab). Statistical differences at each agar concentration were determined using a Mann-Whitney test (*p < 0.0001). Data shown is mean and SD (n = 15). (**b**) Relative motility of rod isolates compared to the average motility of helical isolates decreases with increasing agar concentrations. Data shown is mean and SD (n = 3). All helical (H2, H3, H4) and rod (R2, R3, R4) isolates were also analysed by genome sequencing.

Together, our work demonstrates the susceptibility of *C. jejuni* to transforming into a rod-shaped bacterium. Helical cell shape is maintained by a delicate balance of PG lengths and crosslinking^9^,^10^, which can be disrupted by a single point mutation at numerous locations in the PG carboxypeptidase genes *pgp1* or *pgp2*. Yet despite a propensity of *C. jejuni* to undergo mutations of these genes, the helical morphology remains the dominant form in laboratory and natural environments – reinforcing the importance of helical cell shape to the success of this bacterium. While the possibility of phase variation suggests that the rod *C. jejuni* morphology may have an advantage in certain environments or host associations, various observations from human sources^3^,^52^,^53^ and infection models^54^,^55^ suggest that the ability to cause human campylobacteriosis is most often dependent on a helical *Campylobacter*, a shape that confers the greatest motility (Fig. 6) and enables the colonisation of the cecal crypts.

The PG peptidases necessary for maintaining helical cell shape may offer useful antimicrobial targets to reduce the pathogenicity of *C. jejuni* or decrease its prevalence within the food chain. These targets are broad-spectrum in the sense that endo- and carboxypeptidases are common throughout the bacterial kingdom^56^,^57^, but many PG peptidases are redundant^58^,^59^ and therefore detrimental effects might be compensated in other species and it may also be possible to design narrow-spectrum options that specifically target Pgp1 or Pgp2. Intriguingly, Liu *et al*.^60^ recently reported a bacterial cell shape-determining inhibitor of *H. pylori* Csd4 (homologue of Pgp1 in *C. jejuni*) that causes significant cell straightening of *H. pylori* and a diminished, yet observable, effect on the morphology of *C. jejuni*.

## Methods

### Bacterial strains, media and growth conditions

*C. jejuni* strains were routinely cultured on Mueller Hinton (MH) agar (Oxoid) supplemented with 5% defibrinated horse blood (Oxoid) and 5 μg/ml trimethoprim (Tp). Defined and Tn mutants and complemented strains were selected on 10 μg/ml chloramphenicol (Cm) or 50 μg/ml kanamycin (Km), as appropriate. *Campylobacter* spp. bacterial cultures were grown in standard microaerophilic conditions (5% CO_2_, 5% H_2_, 85% N_2_, 5% O_2_) at 42 °C in a MACS VA500 variable atmosphere work station (Don Whitley Scientific). Electrocompetent *Escherichia coli* and *C. jejuni* used in cloning were prepared and transformed as previously described^61^. Bacterial strains and plasmids used in this study are detailed in Table S5.

### Signature-tagged transposon mutant library

Generation of the *C. jejuni* M1 STM library was described in Grant *et al*.^23^. Briefly, *mariner*-based Tns containing a chloramphenicol acetyl-transferase (*cat*) cassette and a unique 40 bp DNA tag (‘signature tag’) were used in an *in vitro* transposition of WT *C. jejuni* M1 DNA. Transposed DNA was cloned into WT *C. jejuni* M1 by natural transformation. Chloramphenicol-resistant Tn mutant colonies were selected after 60–72 h growth on MH agar, subcultured on fresh agar and stored at −80 °C. Fifty mutants were recovered from each transformation. Mutants were labelled first by their signature tag (1–95). and then by a mutant number (1–50).

### Colony purification by colony morphology

Agar or broth cultures of *C. jejuni* were serially diluted in MH broth, spread onto fresh MH agar and incubated in standard growth conditions. Colonies were inspected by eye after 24–48 h growth and either quantified or isolated based on colony morphology. Helical colonies displayed a shiny and rounded morphology, whereas rod colonies displayed a grey, flat and often larger morphology. Cell morphology was confirmed by transferring bacteria from a colony to a glass slide and viewing cells by light microscopy.

### DNA sequencing

Sanger sequencing was performed by Source BioScience LifeSciences. WGS was performed at the Wellcome Trust Sanger Institute. Isolates were sequenced as multiplex libraries with 100 or 150 bp paired-end reads using next-generation Illumina HiSeq or MiSeq sequencing technology, respectively. For the sequencing of M1cam helical to rod isolates, Sequencing libraries were prepared using the NEBNext Ultra II DNA library prep kit (New England Biolabs). 250 ng DNA was sheared to 400 bp fragments in microTUBE screw-cap tubes in a M220 focused-ultrasonicator (Covaris). Following DNA library preparation, the library size was determined with a Bioanalyzer 2100 (Agilent), quantified using the Qubit dsDNA BR kit (Life Technologies), pooled in equal quantities, and analysed with the NEBNext library quant kit (New England Biolabs). The pooled library was subjected to 150 bp paired-end sequencing (Genomics core facility at Cancer Research UK). The read files were demultiplexed using the demuxFQ tool developed at Cancer Research UK. For WGS analysis, *de novo* draft assemblies of each isolate were created and sequencing reads were mapped to the reference genome for the relevant *C. jejuni* strain using the pipeline described in ref. 62. SNPs and INDELs were called using SAMtools mpileup^63^.

### Analysis of homopolymeric tracts

The length of HTs in PVRs was assessed using the HT length of each sequencing read spanning a defined region. To be counted, the sequencing read must cover the entire region, as determined by the presence of nucleotides on either side of the HT. The distribution of HT lengths at each PVR was converted into a percentage of total reads and compiled based on whether the HT length was in or out of frame with the annotated gene sequence (‘on’ or ‘off’, respectively).

### Recombinant DNA techniques

Standard methods were used for molecular cloning^64^. Chromosomal and plasmid DNA purification, DNA modification and ligations were performed using commercial kits according to the manufacturers’ instructions (QIAGEN, Thermo Scientific, New England Biolabs). DNA concentration was measured using a Nanodrop ND-1000 spectrophotometer (Thermo Scientific). PCR primers were purchased from Sigma (Sigma Aldrich). Thermal cycling was performed in a Gene Amp PCR System 9700 (PE Applied Biosystems) or T100^T^ Thermal Cycler (Bio-Rad). Thermal cycling conditions were 96 °C for 2 min, then 30 cycles at 96 °C for 1 min, 55–60 °C for 1 min and 72 °C for 30 sec/kb, and finally an extension at 72 °C for 5 min.

### Generation of *C. jejuni* defined gene deletion mutants, complemented strains and site-directed mutants

Targeted gene deletions of *CJM1_0159, CJM1_0631* and *dtpT* were performed by exchanging the gene of interest with a *cat* cassette from pRY111^65^. The *cat* cassette was amplified with primers containing *Kpn*I (dare008), *Bam*HI (dare009), *Pst*I (dare010) or *Sac*I (dare011) restriction endonuclease (RE) target sites. Flanking regions of each deleted gene were amplified using upstream and downstream primers (0159_1 to 4; 0631_1 to 4; or dtpT_1 to 4) containing RE sites matched to the chosen *cat* cassette primers. PCR-amplified fragments were ligated to pUC19 prior to transformation into *E. coli*. Purified plasmid DNA was used to naturally transform *C. jejuni*. The correct genomic rearrangements in the resulting *C. jejuni* mutants were confirmed by PCR and sequencing using the primers 0159_ck1 to ck5; 0631_ck1 to ck4; or dtpT_ck1 and ck2, respectively. Primers used in this study are listed in Table S6.

Site-directed mutagenesis of *pgp1* and *pgp2* was performed by amplifying the gene of interest with primers incorporating nucleotide change (s) (pgp1_1 to 6 or pgp2_1 to 6, respectively). Using overlapping sequences, an antibiotic resistance cassette (amplified using primers pgp1_7 and 8 or pgp2_7 and 8, respectively) was incorporated between the mutated gene and the downstream region (amplified using primers pgp1_9 and 10 or pgp2_9 and 10). PCR-amplified fragments with overlapping ends were annealed using a Gibson Assembly^®^ Cloning Kit (New England Biolabs) according to the manufacturer’s instructions and ligated into the pMiniT vector (New England Biolabs) prior to transformation of *E. coli*. Purified plasmid DNA was used to naturally transform *C. jejuni*. The correct genomic rearrangements in the resulting *C. jejuni* mutants were confirmed by PCR and sequencing using the primers pgp1_ck1 to ck3 and pgp2_ck1 to ck3. Bacterial strains and plasmids used in this study are detailed in Table S5. Primers used in this study are listed in Table S6.

### Plasmid rescue

Plasmid rescue was used to assess the chromosomal location of Tns within *C. jejuni* M1 STMs. The technique was based on the method described in Grant *et al*.^23^ but was amended to make use of vector cloning. BglII-digested STM genomic DNA was ligated to BamHI-digested and dephosphorylated pUC19. Ligations were transformed into *E. coli* DH5α (Thermo Scientific) according to manufacturer’s instructions and transferred onto LB agar supplemented with chloramphenicol (Cm). Colonies present after O/N incubation were used to inoculate 5 ml LB broth with Cm. After O/N incubaction of LB cultures, plasmid DNA was extracted and analysed by Sanger sequencing using primers directed out of the Tn (AJG227 and CC1, Table S6).

### Linker PCR

Linker PCR was an alternative method used to assess the chromosomal location of Tns within *C. jejuni* M1 STMs. Linker oligonucleotides (254 and 256, Table S6) were annealed in 1x annealing buffer (100 mM Tris (pH 8), 500 mM NaCl, 10 mM EDTA) at 95 °C for 3 min and cooled at 21 °C for 1 h. The annealed Linker oligonucleotides were ligated to RsaI-digested STM genomic DNA. Ligated Linker-STM DNA fragments were amplified in a PCR using a Tn-specific primer and a Linker-specific primer (CAT15 and 258, respectively, Table S6). The PCR product was resolved by gel electrophoresis and analysed by Sanger sequencing using primers directed out of the Tn (AJG227 and CC1, Table S6).

### Pulsed-field gel electrophoresis

Major chromosomal patterns were compared between WT and STM *C. jejuni* M1 DNA using PFGE. The RE SacII was chosen for DNA fragmentation as SalI, SmaI and KpnI (also used for PFGE of *Campylobacter* species)^24^,^25^,^66^ all had recognition sequences within the Tn^55^. The PFGE method used was based on those described in Rivoal *et al*. and Ribot *et al*.^24^,^25^. WT and STM *C. jejuni* M1 agar cultures, washed and diluted in PBS to an OD_600 nm_ of 0.6–0.8, were preserved and lysed within plugs made with 2% low-melt agarose (Promega) in TE pH 8. Genomic DNA within the agarose plugs was digested with SacII for 5 h at 21 °C, washed in 0.5x TBE and resolved by gel electrophoresis in 1% agarose in 0.5x TBE. Electrophoresis was performed according to manufacturer’s instructions at 6 V/cm for 20 h with a ramped pulse of 5–50 s using CHEF-DR^®^ II Pulsed Field Electrophoresis Systems (Bio-Rad) and connected to a LTD 20 cooling system (Hybaid). Gels were stained with 0.3x SYBR^®^ Safe (Invitrogen) and imaged on a GelDoc^TM^ XR + (Bio-Rad) with Image Lab 3.0 software (Bio-Rad).

### Muropeptide analysis

Peptidoglycan (PG) purification and digestion protocols were adapted from those described in Glauner^67^, Li *et al*.^68^ and Frirdich *et al*.^10^. HPLC of purified and muramidase-digested *C. jejuni* PG was performed in the same manner and using the same instrumentation as described in Christie *et al*.^69^.

### Calculations of mutation frequency and rate

The helical-to-rod switch observed in *C. jejuni* populations was quantified by mutation frequency and mutation rate. Mutation frequency, *f*, was calculated as the proportion of rod-shaped mutants, P_R_, that emerged during exponential growth of spiral isolates^70^. This value was incorporated into Drake’s calculation for a constant rate of mutation^71^:


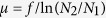


where *μ* is the mutation rate and *N* is the population size at time 1 or 2. We applied these equations to colony counts of helical and rod bacteria, plated on MH agar from liquid cultures of helical *C. jejuni* M1 undergoing exponential growth.

### Light Microscopy

Light microscopy was performed on a Nikon Eclipse E200 light microscope under a 100x objective lens.

### Scanning electron microscopy

To prepare samples for SEM, *C. jejuni* overnight (O/N) agar cultures were resuspended in 1 ml ddH_2_O and pelleted by centrifugation (9,300 × *g*) in microcentrifuge tubes. Bacteria were washed a total of four times before being fixed in 4% paraformaldehyde-1% glutaraldehyde in 0.1 M PBS. Fixed cells were stored at 4 °C prior to microscopy.

### Motility assay

The motility of *C. jejuni* was quantified using motility agar made with 0.4%, 0.6%, 0.8% and 1.0% (w/v) select agar (Sigma) in MH broth. Motility agar was used to fill 6-well plates (7 ml of agar per well) 20 min prior to use. *C. jejuni* isolates were transferred *via* pipette tip from 12 h lawn growth (on MH agar plates) into each well of the motility agar. For each strain to be tested, three replicate 6-well plates were incubated for each motility agar concentration. Motility was measured as the diameter of the halo of motility after 12 h incubation.

### Sequencing data

Genome sequencing data has been deposited in the European Nucleotide Archive (http://www.ebi.ac.uk/ena), study accession PRJEB16677; ERS and ERR accession numbers are provided in Table S7.

## Additional Information

**How to cite this article**: Esson, D. *et al*. Genomic variations leading to alterations in cell morphology of *Campylobacter* spp. *Sci. Rep.* **6**, 38303; doi: 10.1038/srep38303 (2016).

**Publisher’s note:** Springer Nature remains neutral with regard to jurisdictional claims in published maps and institutional affiliations.

## Supplementary Material

Supplementary Table S7

Supplementary Information

## Figures and Tables

**Table 1:** Mutations detected in *pgp1* and *pgp2* in rod-shaped *C. jejuni* isolates.

Base location of change in M1	Nucleotide change	Amino acid change	*C. jejuni* isolates with mutation
1269447 (*pgp1*)	C > T	R91C	51-40, 51-43, 54-40, 54-43, 55-49
1269149 (*pgp1*)	5 A > 4 A	Stop at 205	11168_R1, 11168_R2, 11168_R3
1268944 (*pgp1*)	4 A > 5 A	Stop at 261	19-36 R°, H5_P2_R1
1268939 (*pgp1*)	T > A	V260D	17-19 R°
1268899 (*pgp1*)	5 T > 4 T	Stop at 281	5-45 R*, 17-48 R*
1268857 (*pgp1*)	2 T > T	Stop at 295	38-14 R*
1268827 (*pgp1*)	3 A > 2 A	Stop at 299	17-14 R*
1268739 (*pgp1*)	3 A > 2 A	Stop at 334	17-49*
1268531 (*pgp1*)	8 A > 7 A	Stop at 403	Δ*0159_R*, 19-34, 17-38 R°, 18-26 R*, R1, R2, R3, R4, R5, R6, R7, 81116_R2, 81116_R3, 81-176_R2, 81-176_R3, R’1, R’2, R’3, R’4, R’5, R’6, R’7, R’8, R’9, R’10, R’11, R’12, R’13, R’14, R’15, R’16, R’17, R’18, R’19 m R’20, R’21, R’22, R’23, R’24, R’25, R’26, R’27, R’28, R’29, R’30, R’31, R’32, R’33, R’34, R’35, R’36, R’37, R’38, R’39, R’40, R’41, R’42, R’43, R’44, R’45, R’46, R’47, R’48, R’49, R’51, R’52, R’53, R’54, R’55, R’56, R’57, R’58, R’59, R’60, R’61, R’62, R’63, R’64, R’65, R’66, R’67, R’68, R’69, R’70, R’71, R’72, R’73, R’74, R’75, R’76, R’77, R’78, R’79, R’80, R’81, R’83, R’84, R’85, R’86, R’87, R’89, R’90, R’91, R’92, R’93, R’94, R’95, R’96, R’97, R’98, R’99, R’100, R’101, R’102, R’103, R’104, R’105, R’106, R’107, R’108, R’109, R’110, R’111
	8 A > 9 A	Stop at 408	17-34 Ro, 81116_R1
1268425 (*pgp1*)	ATA > A	Stop at 438	43-4 R*
846702 (*pgp2*)	2 G > 4 G	Stop at 101	R’50, R’82, R’88
846721 (*pgp2*)	C > T	Q93Stop	22-12
846660 (*pgp2*)	C > T	P113L	H5_P2_R2
846319 (*pgp2*)	3 A > 2 A	Stop at 234	H5_P4_R1
846256 (*pgp2*)	4 A > 3 A	Stop at 247	50-1, 50-2, 50-3, 50-6, 50-9, 50-10, 50-18, 50-19, 50-20, 50-34, 51-6, 51-9, 51-31, 51-35, 51-39, 51-41, 51-44, 53-1, 53-9, 53-10, 53-14, 53-17, 53-23, 53-23, 53-26, 53-27, 53-34, 53-35, 53-42, 54-6, 54-26, 54-35, 54-37, 54-44, 54-45, 54-48, 54-49, 54-50, 55-28, 55-29, 55-33, 55-34, 55-38, 55-40, 55-41, 55-42, 55-50, 57-32, 57-33, 57-34, 57-36, 57-37, 57-38, 57-39, 57-40, 57-41 R, 55-35 R
846075 (*pgp2*)	4 T > 5 T	Stop at 312	H2_P1_R1
846037 (*pgp2*)	4 T > 3 T	Stop at 322	62-5 R*
846702 (*pgp2*)	2 G > 4 G	Stop at 101	R’50, R’82, R’88

The base location, nucleotide change and predicted effect on translation of each mutation in *pgp1* and *pgp2* are listed alongside the rod-shaped *C. jejuni* isolates in which each mutation was observed. Isolates include the rod M1 Tn mutants, rod isolates from rod-and-helical M1 Tn mutants (labelled ‘R’), the rod-shaped targeted deletion strain Δ*CJM1_0159::cat* (abbreviated Δ*0159_R*), rod-shaped isolates from the WT M1, 81116, 81-176 and NCTC11168 strains, rod-shaped mutants from serial passages of helical-shaped M1 isolates (*i.e*., H2_P1_R1, where H2 = helical isolate 2, P1 = first passage and R1 = rod 1) and rod-shaped mutants from a colony screen of helical-shaped M1cam. Base locations for all strains are in reference to the *C. jejuni* M1 GenBank genome (CP001900.1). The full-length Pgp1 protein is 464 aa and Pgp2 is 325 aa. All isolates were analysed by Illumina next generation sequencing unless noted; *analysed by Sanger sequencing, °analysed by Sanger sequencing and WGS.

**Table 2:** Prevalence of various lengths of poly-A tract in the *pgp1* allele of *Campylobacter spp.* from various isolation sources.

Poly-A tract length^a^	*C. jejuni*			*C. coli*			Total number of genomes
	Farm chicken	Clinical cases	Other^b^	Farm chicken	Clinical cases	Other^b^	
9 nt	1	18	0	0	1	0	20
8 nt	248	345	64	90	59	24	830
7 nt	2	2	0	1	1	3	9

^a^Length of poly-A at position 1,180 of the *pgp1* gene.

^b^Other sources of isolation comprise wild birds, cattle, ducks, environmental water and unknown sources.
