# Supplementary material for: Genomic variations leading to alterations in cell morphology of Campylobacter spp
Source: Sci Rep. 2016 Dec 2;6:38303. doi: 10.1038/srep38303 (PMC5133587; doi:10.1038/srep38303)
Supplement: Supplementary Information [file srep38303-s2.pdf]

ADDITIONAL INFORMATION

Genomic variations leading to alterations in cell morphology of *Campylobacter* spp.

Running head: Genome changes causing *Campylobacter* shape change

Diane Esson[1], Alison E Mather[2a], Eoin Scanlan[1b], Srishti Gupta[1], Stefan PW de Vries[1], David Bailey[3], Simon R Harris[2], Trevelyan J McKinley[1c], Guillaume Méric[4], Sophia K Berry[1], Pietro Mastroeni[1], Samuel K Sheppard[4], Graham Christie[3], Nicholas R Thomson[2,5], Julian Parkhill[2], Duncan J Maskell[1], Andrew J Grant[1]*

[1]Department of Veterinary Medicine, University of Cambridge, Madingley Road, Cambridge, UK.

[2]Wellcome Trust Sanger Institute, Wellcome Trust Genome Campus, Hinxton, Cambridge, UK.

[3]Department of Chemical Engineering and Biotechnology, University of Cambridge, New Museums Site, Pembroke Street, Cambridge, UK.

[4]The Milner Centre for Evolution, Department of Biology and Biotechnology, University of Bath, Claverton Down, Bath, UK.

[5]The London School of Hygiene and Tropical Medicine, London, UK.

[a]Current address: Department of Veterinary Medicine, University of Cambridge, Madingley Road, Cambridge, UK.

[b]Current address: San Francisco Veterans Affairs Medical Center and the University of California, San Francisco, USA.

[c]Current address: College of Engineering, Mathematics and Physical Sciences, University of Exeter, Treliever Road, Penryn, Cornwall, UK.

*To whom correspondence should be addressed: ajg60@cam.ac.uk

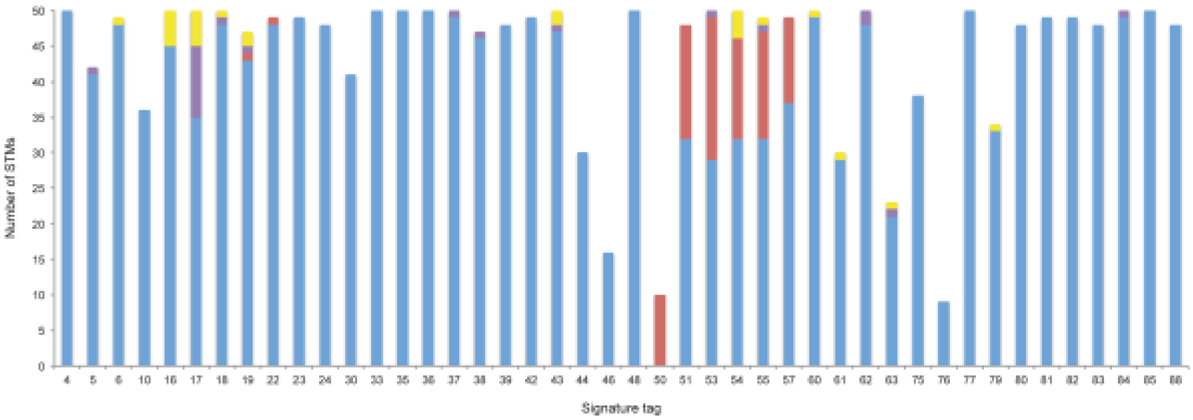

Figure S1 Distribution of observed morphologies within the *C. jejuni* M1 STM Tn mutant library. *C. jejuni* M1 Tn mutants screened by light microscopy are grouped by signature tag. Each signature tag column is colour-coded by the number of Tn mutants of each morphology: helical (blue), rod (red), rod-and-helical (purple) and short helical (yellow). Some non-helical morphologies appear to cluster within select signature tags of the library. Notably, many rod-shaped Tn mutants are from signature tags 50, 51, 53, 54, 55 and 57 and many rod-and-helical Tn mutants are from signature tag 17.

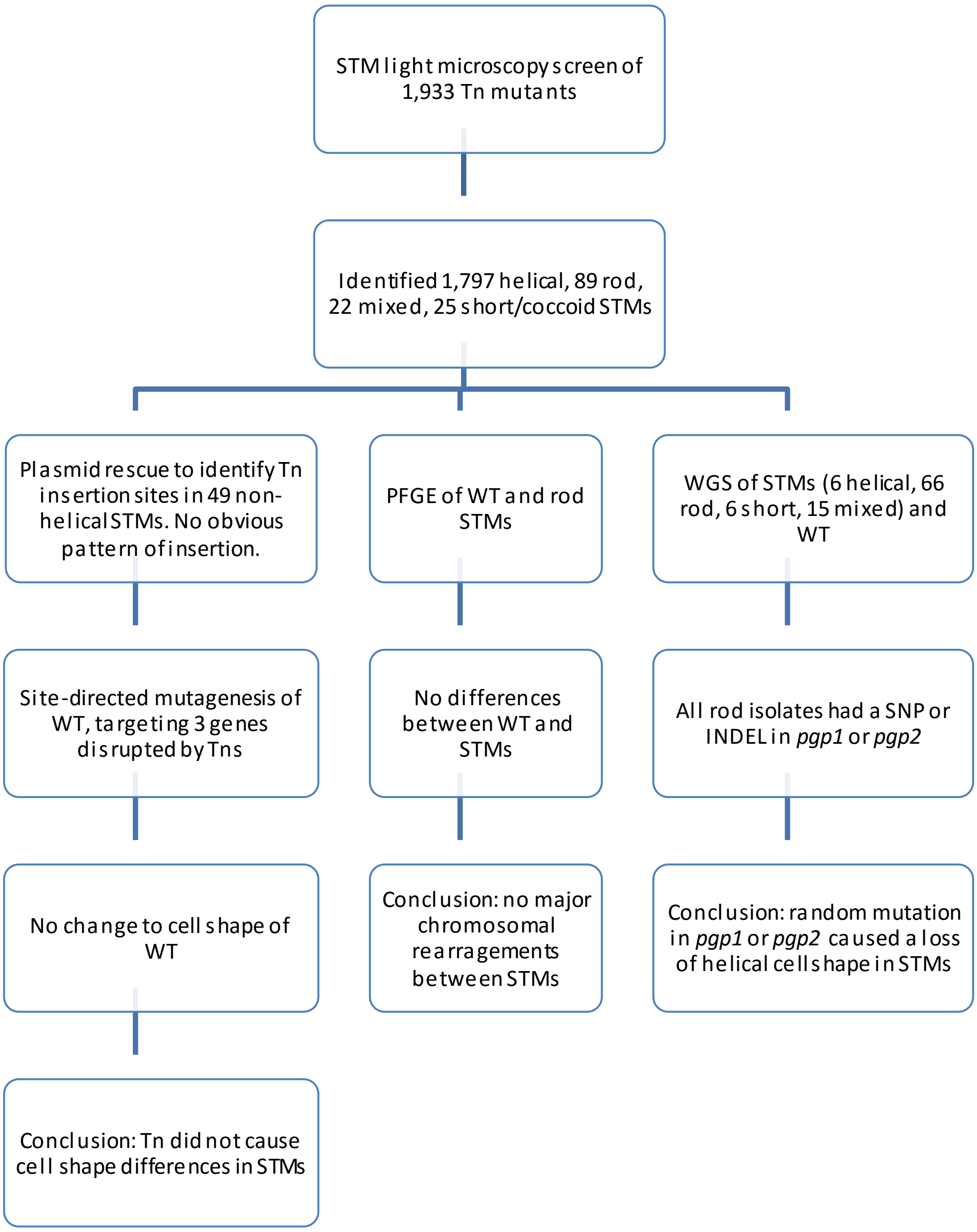

Figure S2 Flow chart detailing the methods applied and conclusions made in the process of the STM analysis.

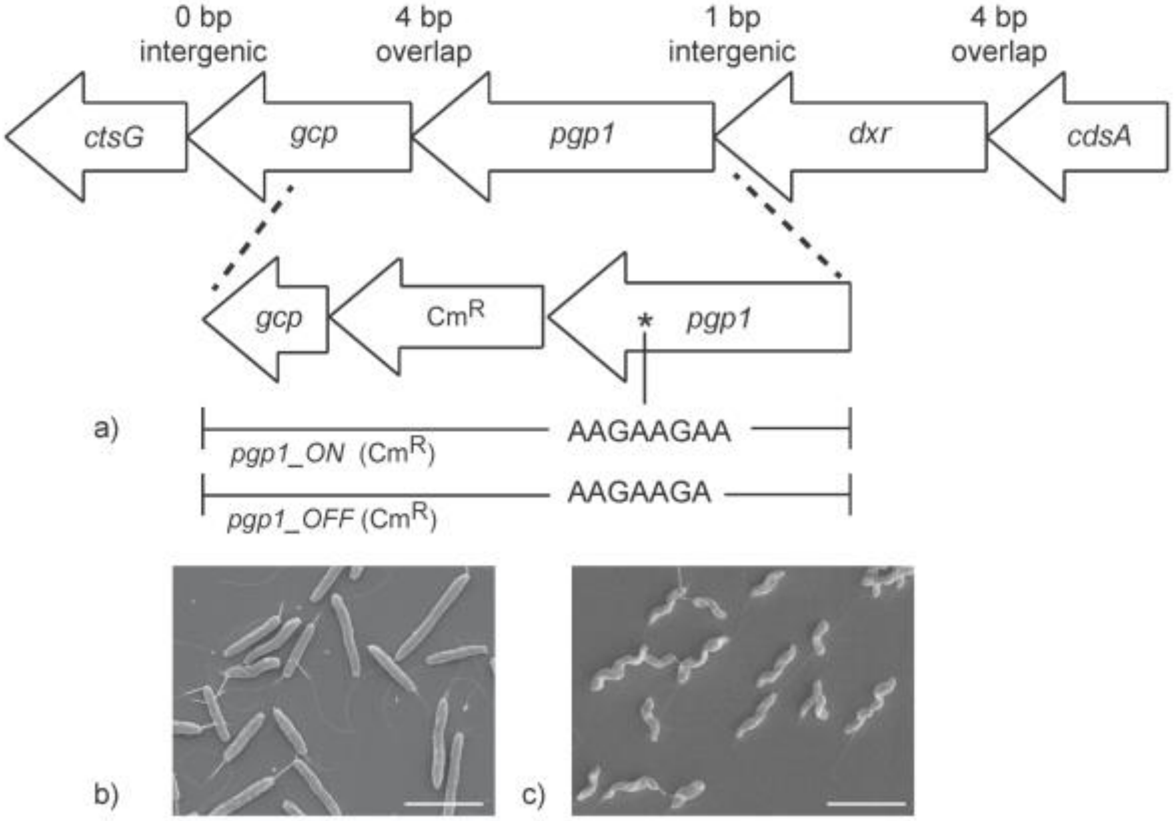

Figure S3 Gene locus and site-directed mutant of *pgp1* in *C. jejuni* M1. (a) The *pgp1* 'locked on' construct exchanged two adenines within the 8-A tract of AT PPVR3 with two guanines, this protected the tract from slipped-strand mispairing but the 'locked on' mutation maintained the same amino acid translation. A *cat* cassette (Cm^R) was incorporated downstream of *pgp1*. The locked 'on' mutation, *cat* cassette and the flanking regions indicated (*pgp1_ON*) were assembled by overlapping PCR and Gibson Assembly®. 'Locked on' constructs were cloned into the suicide vector pMiniT by PCR Cloning (pDARE7), and transformed into (b) rod WT *C. jejuni* M1 to generate (c) the helical *pgp1_ON* strain. Scale bars represent ~2 μm.

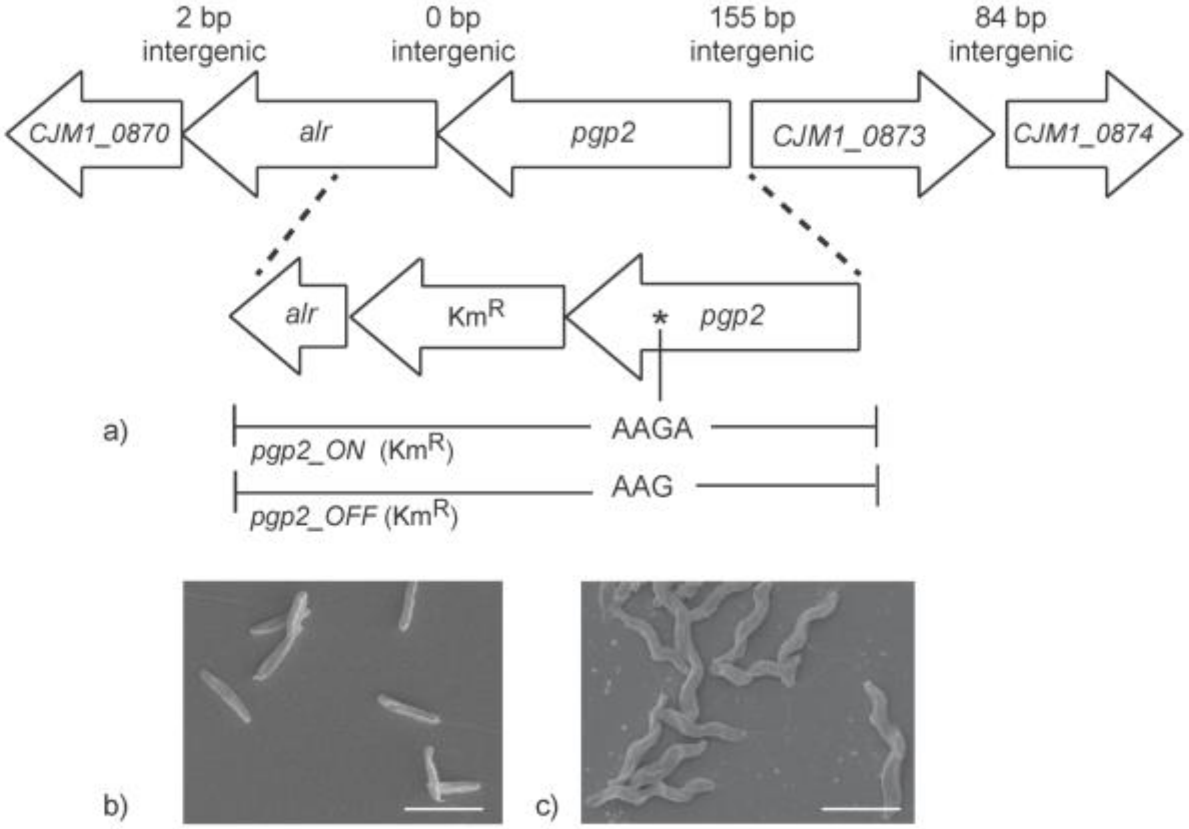

Figure S4 Gene locus and site-directed mutant of *pgp2* in *C. jejuni* M1. (a) The *pgp2* 'locked on' construct exchanged one adenine within the 4-A tract at base location 846256 (CP001900.1) with guanine, this protected the tract from slipped-strand mispairing but the 'locked on' mutation maintained the same amino acid translation. A *cat* cassette (Cm^R) was incorporated downstream of *pgp2*. The locked 'on' mutation, *cat* cassette and the flanking regions indicated (*pgp2_ON*) were assembled by overlapping PCR and Gibson Assembly®. The linear *pgp2_ON* construct was transformed into (b) rod STM 50-18 to generate (c) a helical *pgp2_ON* strain. Scale bars represent ~2 µm.

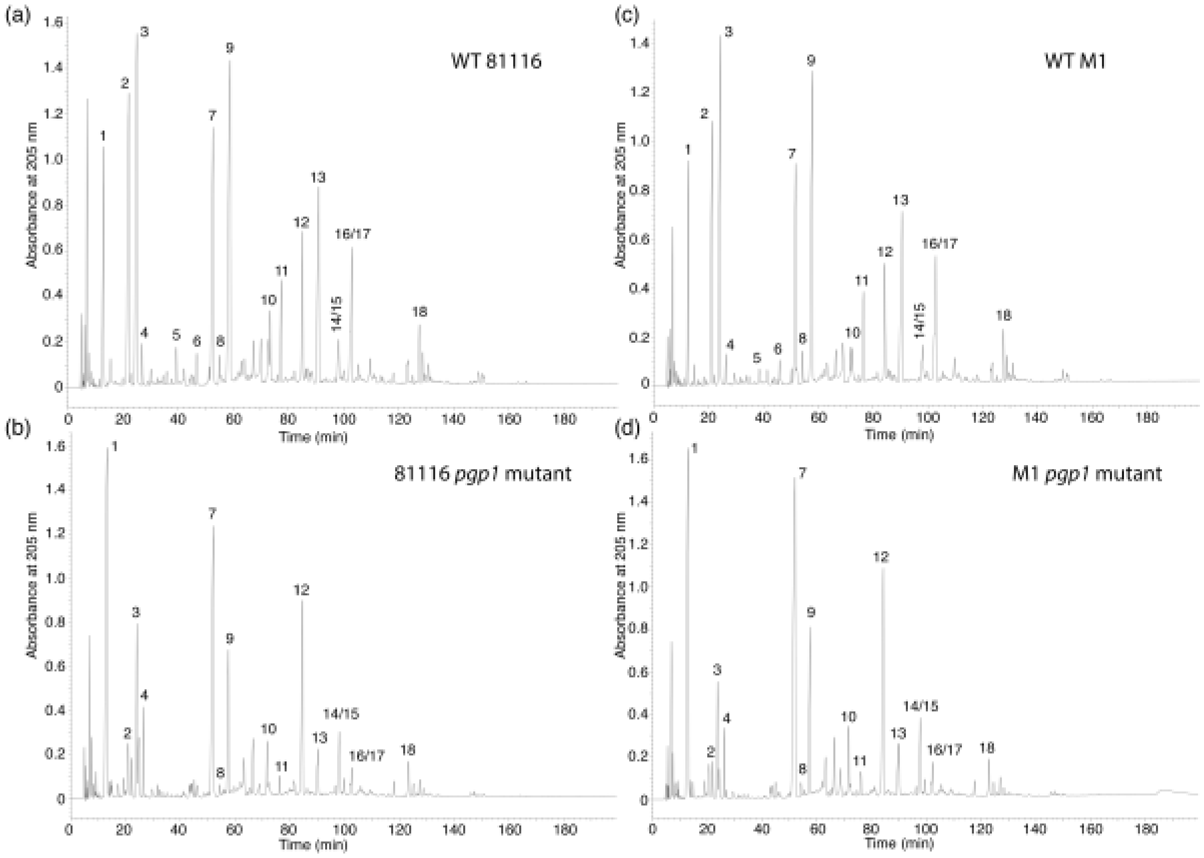

Figure S5 Muropeptide profiles of helical and rod *C. jejuni* 81116 and M1 isolates. HPLC chromatographs of purified PG from (a) WT helical isolate 81116, (b) WT rod isolate 81116_R1 (9A *pgp1* mutant), (c) WT helical isolate M1, and (d) rod isolate M1 STM 43-4_R (INDEL in *pgp1* at base location 1268425). The WT helical isolates (a) and (c) show similar muropeptide profiles. The rod isolates (b) and (d) contain unique mutations in *pgp1* but demonstrate similar muropeptide profiles that sharply contrast with the WT helical profiles. Patterns of peptide length and crosslinking are inferred by the relative absorbance of muropeptide fractions (peaks). Muropeptide peaks have been putatively numbered and identified according to published muropeptide profiles of strain 81-176 (S1).

			Frequency		Rate		Reference
	Organism	Gene or phenotype	Max	Min	Max	Min	
**Spontaneous mutation**	*E. coli*	*lacI*	2.4×10^{-6}		4.5×10^{-7}		(S2)
		Rifampicin^{R}	4.0×10^{-7}	8.0×10^{-9}			(S3)
	H. pylori	Rifampicin^{R}	3.0×10^{-5}	4.0×10^{-8}			(S4)
		Clarithromycin^{R}	1.0×10^{-7}	5.0×10^{-9}			
		Rifampicin^{R}	6.6×10^{-8}	3.8×10^{-8}	1.6×10^{-8}	1.1×10^{-8}	(S5)
		Clarithromycin^{R}	3.0×10^{-9}		8.0×10^{-10}		
	C. jejuni	Nalidixic acid^{R}	4.0×10^{-8}	1.0×10^{-8}			(S6)
		Ciprofloxacin^{R}	2.6×10^{-6}	4.2×10^{-9}			(S7)
			**On-to-Off**	**Off-to-On**	**On-to-Off**	**Off-to-On**	
**Phase variation**	*E. coli*	*ag43*	5.0×10^{-3}	5.0×10^{-4}			(S8)
	C. jejuni	*cj1139-lacZ-cat*	5.3×10^{-2}	5.2×10^{-3}	4.4×10^{-3}	5.8×10^{-4}	(S9)
		cj1139-lacZ-kan	1.0×10^{-2}	6.5×10^{-4}	1.0×10^{-3}	1.0×10^{-4}	
		cj0031	1.3×10^{-2}	1.7×10^{-2}	1.2×10^{-3}	1.8×10^{-3}	
		capA	1.8×10^{-2}	3.6×10^{-2}	1.6×10^{-3}	3.4×10^{-3}	
	C. jejuni	Helical-rod	7.7×10^{-4}		7.25×10^{-5}		This work
	C. jejuni	Helical-rod	2.1×10^{-4}				This work

Table S1 Frequency and rate of morphological change in *C. jejuni* compared to spontaneous mutation and phase variation. The gene or phenotype used to measure mutation in various studies is displayed next to known mutation frequency and/or mutation rate values. Mutation rates are in mutations per division.

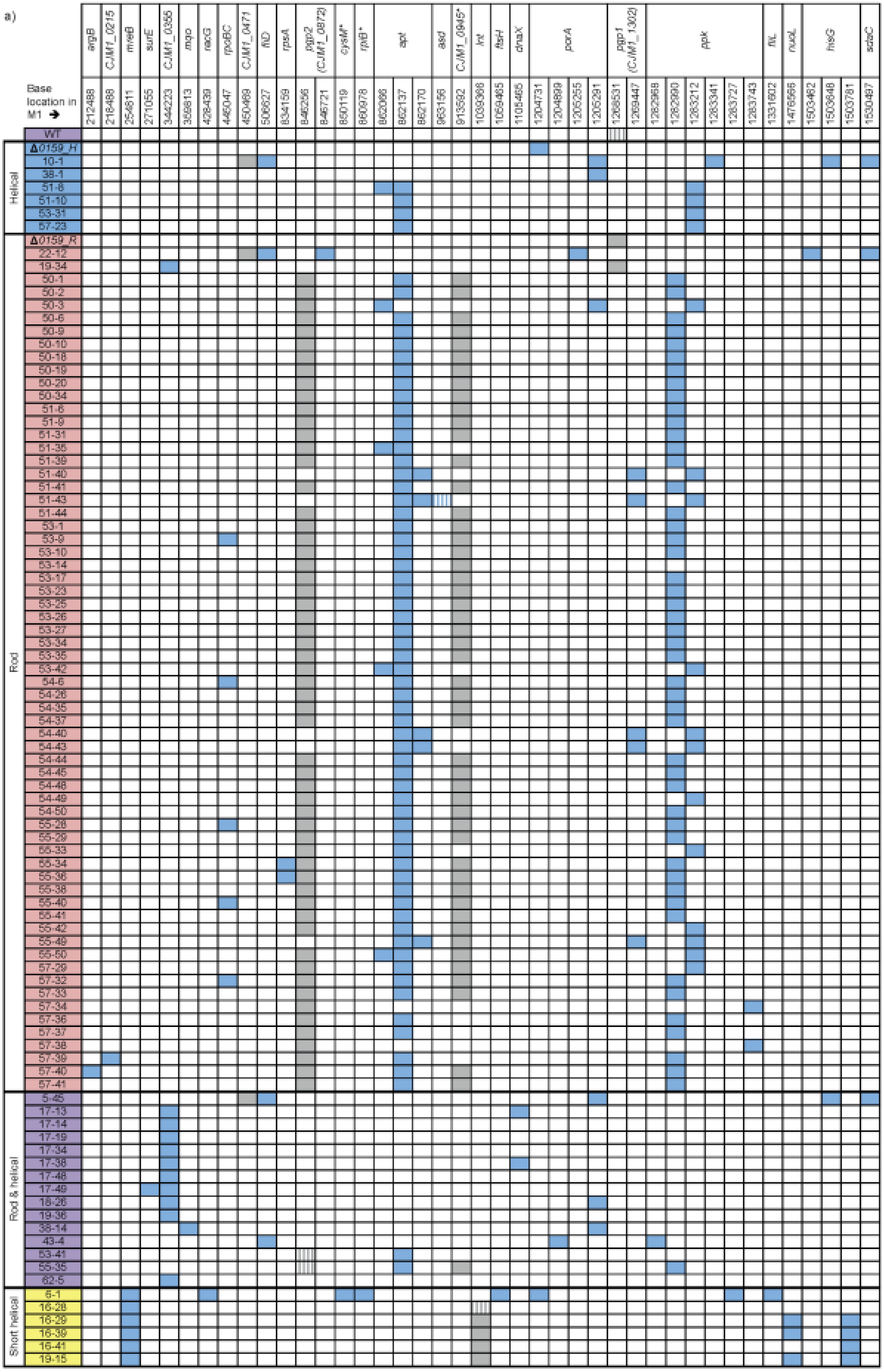
a)
Base location in M1
argB
CJM1_0215
metB
surE
CJM1_0355
mgo
recG
rpoBC
CJM1_0471
fliD
rpsA
pgp2 (CJM1_0872)
cysM*
rplB*
apt
asd
CJM1_0945*
ftsH
dnaX
porA
pgp1 (CJM1_1302)
ppk
fliL
nuoL
hisG
sdaC
212488
218488
254811
271055
344223
355813
428439
445047
450489
506627
834159
846256
846721
850119
860978
862066
862137
862170
863156
913592
1039368
1059465
1105465
1204731
1204899
1205255
1205291
1268531
1269447
1282368
1282390
1283212
1283341
1283727
1283743
1331602
1476566
1503462
1503648
1503781
1530497
WT
Helical
Δ0159_H
10-1
38-1
51-8
51-10
53-31
57-23
Rod
Δ0159_R
22-12
19-34
50-1
50-2
50-3
50-6
50-9
50-10
50-18
50-19
50-20
50-34
51-6
51-9
51-31
51-35
51-39
51-40
51-41
51-43
51-44
53-1
53-9
53-10
53-14
53-17
53-23
53-25
53-26
53-27
53-34
53-35
53-42
54-6
54-26
54-35
54-37
54-40
54-43
54-44
54-45
54-48
54-49
54-50
55-28
55-29
55-33
55-34
55-36
55-38
55-40
55-41
55-42
55-49
55-50
57-29
57-32
57-33
57-34
57-36
57-37
57-38
57-39
57-40
57-41
Rod & helical
5-45
17-13
17-14
17-19
17-34
17-38
17-48
17-49
18-26
19-36
38-14
43-4
53-41
55-35
62-5
Short helical
6-1
16-28
16-29
16-39
16-41
19-15

91

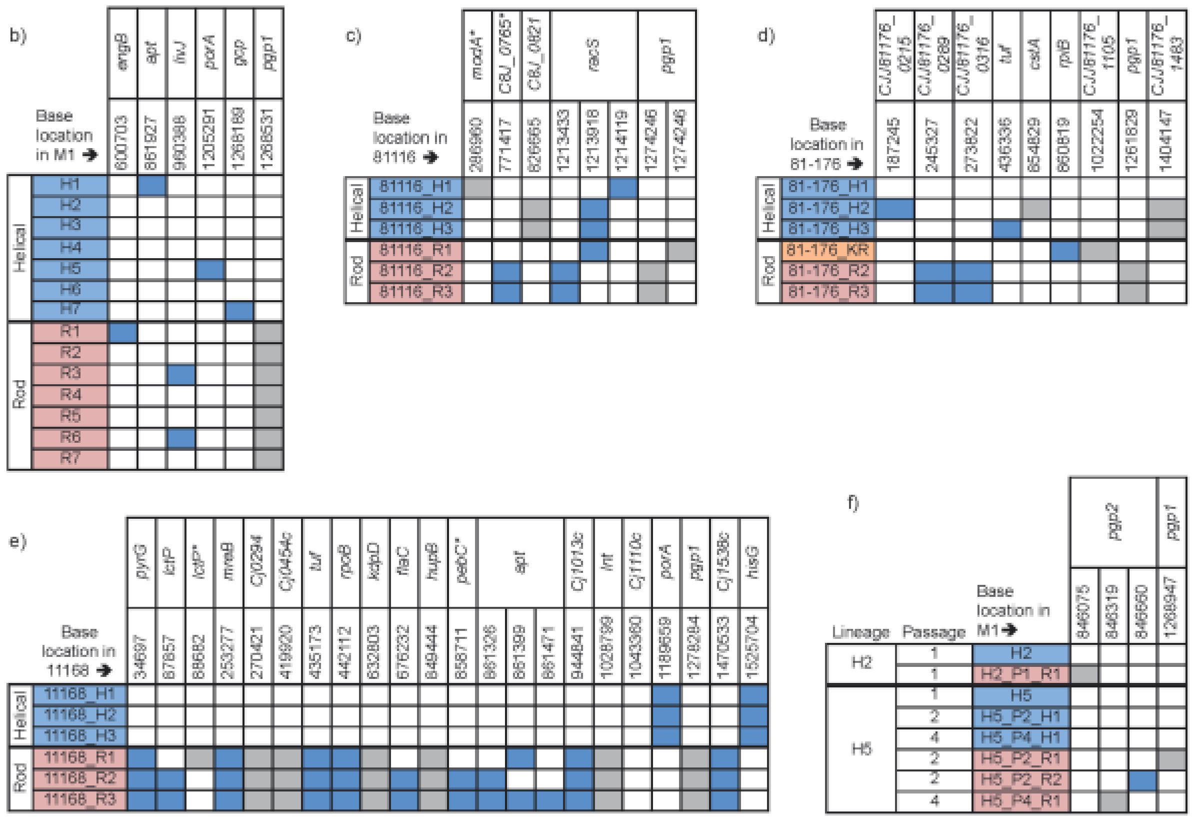

Table S2 SNPs and INDELs detected by genome sequencing in *C. jejuni* strains. SNPs (blue boxes) and INDELs (grey boxes) are organised by gene and base locations within the *C. jejuni* M1 (CP0019001), 81116 (CP000814), 81-176 (CP000538.1), or NCTC11168 (AL111168.1) genomes. Solid fill indicates > 90% of next generation sequencing (NGS) reads contain the variation; striped fill indicates 10-90% of NGS reads contain the variation. Genes with a mutation located upstream of the coding region are represented by an asterisk (*). *C. jejuni* isolates are organized and colour-coded by morphology: helical (blue), rod (red), rod-and-helical (purple), short helical (yellow) and kinked rod (orange). (a) SNPs and INDELs present in *C. jejuni* M1 Tn mutants and targeted deletion strains Δ*CJM1_0159*::*cat_H* (Δ*0159_H*) and Δ*CJM1_0159*::*cat_R* (Δ*0159_R*) that are not present in the WT *C. jejuni* M1 isolate (with the exception of the partial INDEL in *pgp1*). SNPs and INDELs present in helical, rod and kinked rod WT *C. jejuni* isolates from strains (b) M1, (c) 81116, (d) 81-176 and (e) NCTC11168. (f) SNPs and INDELs present in *C. jejuni* M1 helical-to-rod

passage mutants. Rod mutants are from the original helical isolates H2 and H5 after 1, 2 or 4 passages. Helical isolates from each passage are also shown. All rod isolates contain a SNP or INDEL in *pgp1* or *pgp2*.

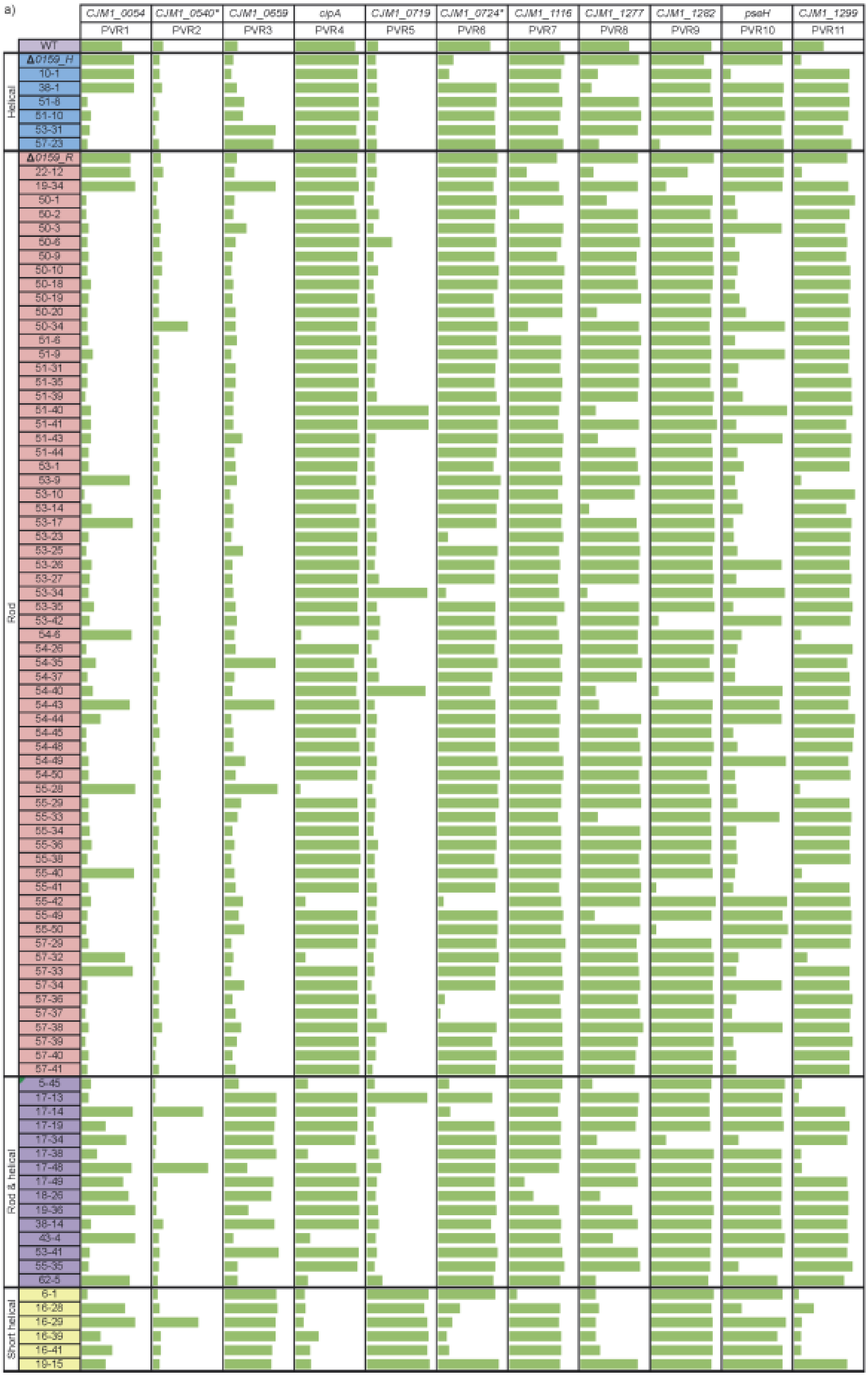
a)
CJM1_0054
CJM1_0540*
CJM1_0659
clpA
CJM1_0719
CJM1_0724*
CJM1_1116
CJM1_1277
CJM1_1282
pseH
CJM1_1299
PVR1
PVR2
PVR3
PVR4
PVR5
PVR6
PVR7
PVR8
PVR9
PVR10
PVR11
WT
Helical
Δ0159_H
10-1
38-1
51-8
51-10
53-31
57-23
Rod
Δ0159_R
22-12
19-34
50-1
50-2
50-3
50-6
50-9
50-10
50-18
50-19
50-20
50-34
51-6
51-9
51-31
51-35
51-39
51-40
51-41
51-43
51-44
53-1
53-9
53-10
53-14
53-17
53-23
53-25
53-26
53-27
53-34
53-35
53-42
54-6
54-26
54-35
54-37
54-40
54-43
54-44
54-45
54-48
54-49
54-50
55-28
55-29
55-33
55-34
55-36
55-38
55-40
55-41
55-42
55-49
55-50
57-29
57-32
57-33
57-34
57-36
57-37
57-38
57-39
57-40
57-41
Rod & helical
5-45
17-13
17-14
17-19
17-34
17-38
17-48
17-49
18-26
19-36
38-14
43-4
53-41
55-35
62-5
Short helical
6-1
16-28
16-29
16-39
16-41
19-15

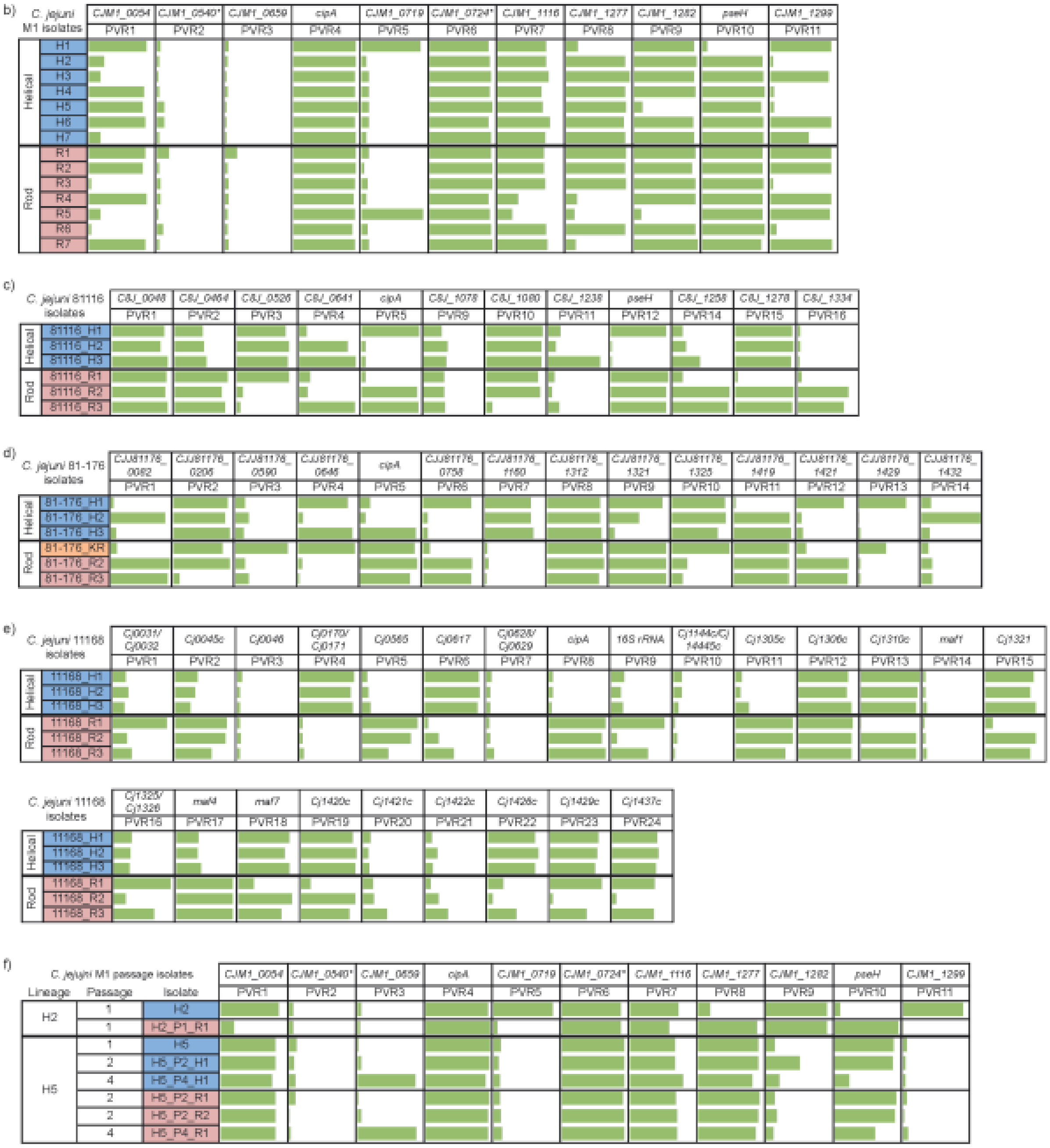

Table S3 Percentage of genome sequence reads demonstrating ‘on’ tract lengths of PVRs. PVRs are defined as G:C homopolymeric tracts seven or more bases in length. PVRs are organised by gene location and genes with PVRs located upstream are indicated with an asterisk (*). *C. jejuni* isolates are organised and colour-coded by morphology: helical (blue), rod (red), rod-and-helical (purple), short helical (yellow) and kinked rod (orange). A PVR is considered ‘on’ if its tract length is in-frame with the

rest of the translated sequence, producing the full-length protein. Percentage of ‘on’ tract lengths are proportionate to the length of the green bars (column width represents 100%). Percentage of ‘off’ tract lengths at each PVR may be inferred by the remaining column width not filled with green. (a) PVRs of *C. jejuni* M1 STMs and targeted deletion strains Δ*CJM1_0159*::*cat_H* (Δ*0159_H*) and Δ*CJM1_0159*::*cat_R* (Δ*0159_R*). PVRs of helical, rod and kinked rod WT *C. jejuni* isolates from strains (b) M1, (c) 81116, (d) 81-176 and (e) NCTC11168. (f) PVRs of *C. jejuni* M1 helical-to-rod passage strains. Rod strains are from the original helical isolates H2 and H5 after 1, 2 or 4 passages. Helical isolates from each passage are also shown.

Peak no.	Muropeptide fraction		Peak no.	Muropeptide fraction	
1	Tri	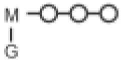	11	Tetra-Tetra-Tri	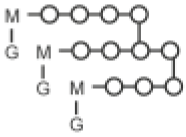
2	Tetra	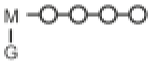	12	Tetra-Tetra-Ac	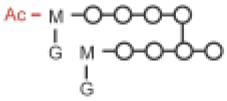
3	Di	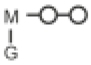	13	Tetra-Tetra-Tetra	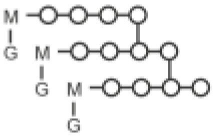
4	Tri-Ac	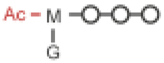	14	Tetra-Tri-Anh I	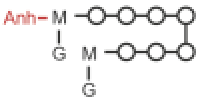
5	Tetra-Ac	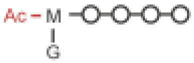	15	Tetra-Tri-Anh II	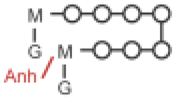
6	Di-Ac	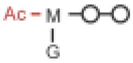	16	Tetra-Tetra-Anh I	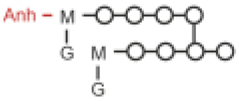
7	Tetra-Tri	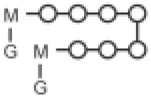	17	Tetra-Tetra-Anh II	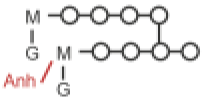
8	Penta-Gly	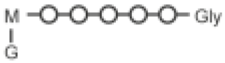	18	Tetra-Tetra-Tetra-Anh I	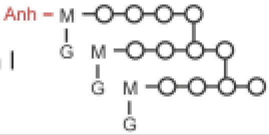
9	Tetra-tetra	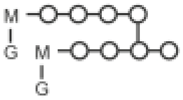			
10	Tetra-Tri-Ac	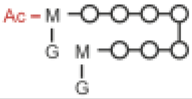			

Table S4 Muropeptide fractions of HPLC peaks. Lengths of each peptide monomer (di-, tri-, tetra- or pentapeptide) are represented with circles. Crosslinked peptides in the dimers and trimers (peaks 7, 9 and 10-18) are joined by dashes. The glycan backbone residues, *N*-acetylmuramic acid (M) and *N*-acetylglucosamine (G), may be acetylated (Ac) or anhydrous (Anh) as indicated. Table adapted from Frirdich *et al* (S1).

**Strain or plasmid**	**Relevant genotype or description**	**Source and/or reference**
C. jejuni M1	Chicken and human clinical isolate (Helical)	Diane Newell, (S10)
C. jejuni M1cam	Derivative of original M1 isolate (Helical)	(S11)
C. jejuni 81-176	Human clinical isolate, hyperinvasive (Helical)	(S12)
C. jejuni 81116	Human clinical isolate (Helical)	(S13)
C. jejuni NCTC11168	Human clinical isolate (Helical)	(S14)
Δ*CJM1_0159_H*	M1 background, Δ*CJM1_0159*, Cm^R (Helical)	This study
Δ*CJM1_0159_R*	M1 background, Δ*CJM1_0159*, Cm^R (Rod)	This study
Δ*CJM1_0631*	M1 background, Δ*CJM1_0631*, Cm^R (Helical and rod)	This study
Δ*dtpT*	M1 background, Δ*dtpT*, Cm^R (Helical and rod)	This study
pgp1_ON	Rod M1 background, pgp1_ON, Cm^R (Helical)	This study
pgp2_ON	Helical M1 background, pgp2_ON, Cm^R (Helical)	This study
22-12_pgp2_ON	Rod M1 STM 22-12 background, *pgp2_ON*, Cm^R, Km^R (Helical)	This study
50-18_pgp2_ON	Rod M1 STM 50-18 background, *pgp2_ON*, Cm^R, Km^R (Helical)	This study
51-44_pgp2_ON	Rod M1 STM 51-44 background+, *pgp2_ON*, Cm^R, Km^R (Helical)	This study
E. coli DH5α	Subcloning Efficiency™ DH5□™ Competent Cells. F$^-$ Φ80*lac*ZΔM15 Δ(*lac*ZYA-*arg*F) U169 *rec*A1 *end*A1 *hsd*R17(r_k^-, m_k^+) *pho*A *sup*E44 *thi*-1 *gyr*A96 *rel*A1 λ$^-$	Thermo Scientific
**Plasmids**		
pUC19	*E. coli* cloning vector, *C. jejuni* suicide vector, Ap^R	New England Biolabs, (S15)
pCE107/70	*C. jejuni* shuttle vector, Km^R	(S16)
pRY111	Source of *Campylobacter cat* cassette, Cm^R	(S17)
pMiniT	*E. coli* cloning vector, *C. jejuni* suicide vector, Ap^R	New England Biolabs
pDARE1	pUC19 derivative encoding Δ*CJM1_0159,* Ap^R, Cm^R	This study
pDARE2	pCE107/70 derivative encoding *CJM1_0159,* Km^R	This study
pDARE3	pUC19 derivative encoding Δ*CJM1_0631,* Ap^R, Cm^R	This study
pDARE4	pCE107/70 derivative encoding *CJM1_0631,* Km^R	This study
pDARE5	pUC19 derivative encoding Δ*dtpT,* Ap^R, Cm^R	This study
pDARE6	pCE107/70 derivative encoding *dtpT,* Ap^R, Cm^R	This study
pDARE7	pMiniT derivative encoding *pgp1_ON,* Ap^R, Km^R	This study
pDARE8	pMiniT derivative encoding *pgp1_OFF,* Ap^R, Cm^R	This study
pDARE9	pMiniT derivative encoding *pgp2_OFF,* Ap^R, Cm^R	This study

Table S5 Bacterial strains and plasmids used in this study. Abbreviations for antibiotics: Cm, Chloramphenicol; Km, Kanamycin; Ap, Ampicillin.

Primer	Target	Sequence (5' – 3')
dare008	*cat* cassette	gaattcggtaccCTCGGCGGTGTTCCTTTCCAAGTT
dare009	*cat* cassette	gcatgcggatccCGCCCTTTAGTTCCTAAAGGGTT
dare010	*cat* cassette	gcatgcctgcagCGCCCTTTAGTTCCTAAAGGGTT
dare011	*cat* cassette	agtactgagctcCTCGGCGGTGTTCCTTTCCAAGTT
0159_1	*CJM1_0159* upstream	ggatccgagctcATGCAAAAAGCTAAAATTTTAATTGCC
0159_2	*CJM1_0159* upstream	aagcttggtaccTTTTATAAATATCTTTTCTATTAAAAAAGGC
0159_3	*CJM1_0159* downstream	cccggggatccCTTTTCTTAAATTAATCTCTTTTTTATG
0159_4	*CJM1_0159* downstream	ctcgaggcatgcCTAATCTTGACTTAAAAGCAGTTTTTG
0159_ck1	Δ*CJM1_0159*	AAGAACAGGCTATGCACAAGG
0159_ck2	Δ*CJM1_0159*	ACCTGATTTTGCGATTTTGG
0159_ck3	Δ*CJM1_0159*	GTTTTAATGGCCGTGCTGTT
0159_ck4	Δ*CJM1_0159*	AAGCCTTGCCAGGATCACTA
0159_ck5	Δ*CJM1_0159*	TGCAAATGCAGCCTTAGTTG
0631_1	*CJM1_0631* upstream	ggatccgagctcATGAATAAAATAGAAGTATTTTTGCA
0631_2	*CJM1_0631* upstream	aagcttggtaccTTCCCAATACCTTTCTTTTTTATGCTTA
0631_3	*CJM1_0631* downstream	cccggggatccACTTAAAATACAAAATACACCAAAG
0631_4	*CJM1_0631* downstream	ctcgaggcatgcCTACAAAAGCAGGAAAATTAAAGC
0631_ck1	Δ*CJM1_0631*	TCATGGCTTAATTCCAATGATTCT
0631_ck2	Δ*CJM1_0631*	CCAAAATGCCATCCGTATTC
0631_ck3	Δ*CJM1_0631*	TAGTGGTCGAAATACTCTTTTCGTG
0631_ck4	Δ*CJM1_0631*	CCCTTATCGATTCAAGTGCATCATG
dtpT_1	*dtpT* upstream	ggatccgagctcACAAAGTTATACATAT
dtpT_2	*dtpT* upstream	aagcttggtaccATAAAAAAGAGTAAAACCACAG
dtpT_3	*dtpT* downstream	cccgggctgcagTATTAAGGAGAAAATATGA
dtpT_4	*dtpT* downstream	ctcgaggcatgcTCAAGCTCCATGATGATTTC
dtpT_ck1	Δ*dtpT*	TGCATGTGGATGCTTTTTGT
dtpT_ck2	Δ*dtpT*	AAAGCGGTCATTTTTAAGG
pgp1_1	*pgp1* left flank	ATGAAATTTTTTTAACAATTTTAT
pgp1_2	*pgp1_ON* left flank	TATTCTTCTTATGAACTAAAATACCACTTTCATC
pgp1_3	*pgp1_OFF* left flank	TATCTTCTTATGAACTAAAATACCACTTTCATC
pgp1_4	*pgp1_ON* right flank	ATAAGAAGAATATGCAAACACAATACTCTTTA
pgp1_5	*pgp1_OFF* right flank	ATAAGAAGATATGCAAACACAATACTCTTTA
pgp1_6	*pgp1* right flank	ATCAATCTTTGTGAATTGCATCATTCAAATTCCACTAAAATAG
pgp1_7	*cat* cassette	CTATTTTAGTGGAATTTGAATGATGCAATTCACAAAGATTGAT

pgp1_8	*cat* cassette	CTAGGATAAGATTTTTCATTTATTTATTCAGCAAGTCTTGT
pgp1_9	*pgp1* dow nstream	ACAAGACTTGCTGAATAAATAAATGAAAAATCTTATCCTAG
pgp1_10	*pgp1* dow nstream	CGCACTGCATTTTTAAGTCC
pgp1_ck1	*pgp1_ON* and *pgp1_OFF*	TGCTGCTAATGAAGTTGGTGT
pgp1_ck2	*pgp1_ON* and *pgp1_OFF*	CCGCCTACCAACGCCAAAATT
pgp1_ck3	*pgp1_ON* and *pgp1_OFF*	GCAATGAAACCACCGTTCCT
pgp2_1	*pgp2* left flank	gagctcggtaccTGCCTAAAAAAGACATTCCTATAAA
pgp2_2	*pgp2_ON* left flank	CTGTTCAAATTTCATCTTATCAAAAC
pgp2_3	*pgp2_OFF* left flank	CTGTTCAAATTTCACTTATCAAAAC
pgp2_4	*pgp2_ON* right flank	AAACGTTTTGATAAGATGAAAT
pgp2_5	*pgp2_OFF* right flank	AAACGTTTTGATAAGTGAAAT
pgp2_6	*pgp2* right flank	GATATTCTCATTTTAGCCATTTATTGCTCTGCTAAAAT
pgp2_7	Km^R cassette	ATTTTAGCAGAGCAATAAATGGCTAAAATGAGAATATC
pgp2_8	Km^R cassette	GAATTTTTATTAAAGACATTTATTGCTCTGCCTAAAACAATTCATCCAG
pgp2_9	*pgp2* dow nstream	CTGGATGAATTGTTTTAGGCAGAGCAATAAATGTCTTTAATAAAAATTG
pgp2_10	*pgp2* dow nstream	gaattctctagaGAATAAGATCTATAGCATGTTC
pgp2_ck1	*pgp2_ON* and *pgp2_OFF*	GGTTTTGGGTAGGCTGAAACT
pgp2_ck2	*pgp2_ON* and *pgp2_OFF*	TCCATCCATTTCATCTGCACT
pgp2_ck3	*pgp2_ON* and *pgp2_OFF*	ACATGGAAACTAGCTTGGACA
AJG227	Transposon	CCCGGGAATCATTTGAAG
CC1	Transposon	TAACTTGGAAAGGAACACCG
254	Linker	CGACTGGACCTGGA
256	Linker	GATAAGCAGGGATCGGAACCTCCAGGTCCAGTCG
258	Linker	GATAAGCAGGGATCGGAACC
CAT15	*Cat* cassette	TAGTGGTCGAAATACTCTTTTCGTG

Table S6 Primer sequences used in this study. Upper-case indicates homology to target sequence. Restriction enzyme sites are underlined and preceded by an arbitrary 6-bp sequence.

Supplemental references

S1. Frirdich, E. *et al.* Peptidoglycan-modifying enzyme Pgp1 is required for helical cell shape and pathogenicity traits in *Campylobacter jejuni*. PLoS. Pathog. **8**, e1002602 (2012).

S2. Drake, J.W., Charlesworth, B., Charlesworth, D., & Crow, J.F. Rates of spontaneous mutation. *Genetics*. **148**, 1667-1687 (1990).

S3. Baquero, M.R. *et al.* Polymorphic mutation frequencies in *Escherichia coli*: emergence of weak mutators in clinical isolates. *J. Bacteriol.* **186**, 5538-5542 (2004).

S4. Bjorkholm, B., Sjolund, M., Falk, P.G., Berg, O.G., Engstrand, L., & Andersson, D.I. Mutation frequency and biological cost of antibiotic resistance in *Helicobacter pylori*. *Proc. Natl. Acad. Sci. USA.* **98**, 14607-14612 (2001).

S5. Wang, G., Wilson, T.J.M., Jiang, Q., & Taylor, D.E. 2001. Spontaneous mutations that confer antibiotic resistance in *Helicobacter pylori*. *Antimicrob. Agents. Chemother.* **45**, 727-733 (2001).

S6. Gaasbeek, E.J. *et al.* Functional characterization of excision repair and RecA-dependent recombinational DNA repair in *Campylobacter jejuni*. *J. Bacteriol.* **191**, 3785-3793 (2009).

S7. Hanninen, M.L., & Hannula, M. Spontaneous mutation frequency and emergence of ciprofloxacin resistance in *Campylobacter jejuni* and *Campylobacter coli*. *J. Antimicrob. Chemother.* **60**, 1251-1257 (2007).

S8. Chauhan, A., Sakamoto, C., Ghigo, J-M., & Beloin, C. Did I pick the right colony? Pitfalls in the study of the regulation of phase variable antigen 43 adhesin. *PLoS. One.* **8**, e73568 (2013).

S9. Bayliss, C.D., *et al.* Phase variable genes of *Campylobacter jejuni* exhibit high mutation rates and specific mutational patterns but mutability is not the major determinant of population structure during host colonization. Nucleic. Acids. Res. **40**, 5876-5889 (2012).

S10. Friis, C. *et al.* Genomic Characterization of *Campylobacter jejuni* Strain M1. *PLoS. One.* **5**, e12253 (2010).

S11. de Vries SP *et al.* Motility defects in *Campylobacter jejuni* defined gene deletion mutants caused by second-site mutations. *Microbiology.* **161**, 2316-2327 (2015).

S12. Korlath, J.A., Osterholm, M.T., Judy, L.A., Forfang, J.C., & Robinson, R.A. A point-source outbreak of Campylobacteriosis associated with consumption of raw milk. *J. Infect. Dis.* **152,** 592-596 (1985).

S13. Palmer, S.R. *et al.* Water-borne outbreak of *Campylobacter* gastroenteritis. *Lancet.* **1**, 287-290 (1983).

S14. Skirrow, M.B. *Campylobacter* enteritis: a “new” disease. *Br. Med. J.* **2**, 9-11 (1977).

S15. Yanisch-Perron, C. Vieira, J. & Messing, J. Improved M13 phage cloning vectors and host strains: nucleotide sequences of the M13mpl8. *Gene.* **33**, 103-119 (1985).

S16. Larsen, J.C., Szymanski, C., & Guerry, P. N-linked protein glycosylation is required for full competence in *Campylobacter jejuni* 81-176. *J. Bacteriol.* **186**, 6508-6514 (2004).

S17. Yao, R., Alm, R.A., Trust, T.J., & Guerry, P. Construction of new *Campylobacter* cloning vectors and a new mutational cat cassette. *Gene.* **130**, 127-130 (1993).
